# A novel optogenetically tunable frequency modulating oscillator

**DOI:** 10.1371/journal.pone.0183242

**Published:** 2018-02-01

**Authors:** Tarun Mahajan, Kshitij Rai

**Affiliations:** 1 Department of Electrical Engineering, Indian Institute of Technology Delhi, New Delhi, India; 2 Department of Biochemical Engineering and Biotechnology, Indian Institute of Technology Delhi, New Delhi, India; Georgia State University, UNITED STATES

## Abstract

Synthetic biology has enabled the creation of biological reconfigurable circuits, which perform multiple functions monopolizing a single biological machine; Such a system can switch between different behaviours in response to environmental cues. Previous work has demonstrated switchable dynamical behaviour employing reconfigurable logic gate genetic networks. Here we describe a computational framework for reconfigurable circuits in *E*.*coli* using combinations of logic gates, and also propose the biological implementation. The proposed system is an oscillator that can exhibit tunability of frequency and amplitude of oscillations. Further, the frequency of operation can be changed optogenetically. Insilico analysis revealed that two-component light systems, in response to light within a frequency range, can be used for modulating the frequency of the oscillator or stopping the oscillations altogether. Computational modelling reveals that mixing two colonies of *E*.*coli* oscillating at different frequencies generates spatial beat patterns. Further, we show that these oscillations more robustly respond to input perturbations compared to the base oscillator, to which the proposed oscillator is a modification. Compared to the base oscillator, the proposed system shows faster synchronization in a colony of cells for a larger region of the parameter space. Additionally, the proposed oscillator also exhibits lesser synchronization error in the transient period after input perturbations. This provides a strong basis for the construction of synthetic reconfigurable circuits in bacteria and other organisms, which can be scaled up to perform functions in the field of time dependent drug delivery with tunable dosages, and sets the stage for further development of circuits with synchronized population level behaviour.

## Introduction

The aim of synthetic biology is to design and synthesize biological networks that perform desired functions in a predictable manner. These systems then, can be used in conjunction, and be joint to one another to solve a particular real world problem. This is mostly achieved using a combination of genetic logic gates [1–6] which use the property of biological parts being analogous to digital logic within certain defined threshold values, which are similar to the logic gates designed and used in electrical engineering. Several circuits in synthetic biology have been designed to work as expected using these combinations of digital logic to create circuits with applications in the field of pharmaceuticals [7], biofuels [8], to encode memory in cells [9–12] or to create biological analogues of electrical circuits such as transistors [13, 14]. A significant chunk of work in the field synthetic biology has targetted the construction of two important types of networks, namely switches [15, 16] and oscillators [17, 18]. The oscillator is crucial to and lies at the center of this paper. Some of the common topologies which are known to show oscillatory behavior are [17, 19]:

- Goodwin Oscillator [20]
- Repressilator [21]
- Amplified negative feedback oscillator

1. Goodwin Oscillator—This was the first synthetic genetic oscillator to be demonstrated. It consists of a single gene with negative auto regulation, and had three equations in a simple model, for the mRNA, protein and inhibitor concentrations. This laid the framework for oscillations, as oscillations occur in the Goodwin oscillator if hill kinetics is followed with a high cooperativity coefficient [22]. The hill function approximation was shown to be valid if the quasi steady state assumption for the multisite phosphorylation of the transcriptional repressor was made.
2. Repressilator—The repressilator was the first complete ring oscillator that was demonstrated, and was a three component system, with each component repressing the next (A represses B represses C represses A). This circuit was shown to have oscillatory behaviour in silico and in vivo in E.coli, triggering a large amount of work in the field of synthetic biology, and oscillators in particular. The repressilator however, was not a synchronised oscillator, and since then, further two component oscillators such as the Danino oscillator [23] have shown synchronisation in the oscillations of a colony of 200 cells, while the Prindle oscillator [24] has shown gas-phase redox signaling between colonies to create an LCD like display among colonies that are physically separated from each other.
3. Amplified negative feedback oscillator—Leaving aside the biological implementation, this abstract topology is the one used in the Danino Oscillator. This architecture consists of a two component system, with an activator gene (A) activating its own repressor gene (B). In the Danino oscillator there is a latent phase when both the activator and repressor accumulate, thus allowing large amplitudes to be achieved for this configuration. The qualitative behavior of this oscillator is discussed in the modelling section using the mathematical model suggested in [23]. The advantage of the Danino oscillator topology over this amplified negative feedback oscillator is that it uses the small molecule AHL coupled to a constitutively expressed LuxR complex as the activator. This AHL molecule can diffuse across the cell membrane of various cells, where it can complex with the intracellular LuxR, and activate transcription inside the cell. Thus, this provides a means for cell-cell communication, which allows for synchronization of oscillations in small populations of cells over time.

Due to the limited number of parts that have been discovered and properly characterised functionally, a lot of these circuits use the same parts. For instance, the repressilator used the only three classes of repressors that have been properly characterised (LacI, TetR and cI), while the first synthetic bi-stable toggle switch also used two of the repressors from the same list. Consequentially, when we talk of scaling up these circuits, a limitation arises due to the the fact that the same repressors and activators are used in a multitude of circuits in synthetic biology. Thus, we often cannot have two circuits with completely different functionalities present in the same cell, separated from one another and acting independently. Thus, for combining two circuits in one cell with switchability between the two when we desire, we need the circuits to be independent of each other in the sense that the repressors/activators for one system should not interact with the circuitry of the other. This is what is called orthogonality in synthetic biology systems [25].

In particular, consider the case of the repressilator [21] and the genetic toggle switch [15]. The repressilator used the only three classes of repressors that have been properly characterised (LacI, TetR and cI), while the genetic bi-stable toggle switch also used two of the repressors from the same list. Thus, it is not possible to add the two systems in a single cell in tandem and expect them to work independently of each other. This is due to the lack of availability of orthogonal parts that are well defined, characterized, and largely similar in functional form to the one desired or predicted from the model [26].

While there are significant advances being made and libraries of orthogonal parts for use in synthetic genetic circuits are being made [1], the use of these parts remains limited, and there is a need for combining the two circuits in a manner that while working with one aspect of the system in the circuit, the other one remains unaffected. Also there is a need to combine these circuits in such a manner that we can channel between different circuits as required. From here arises the concept of reconfigurability in biological circuits, ie circuits with switchable behaviour. These systems would be the ideal multi-taskers, switching between functionalities as the need arises. Synthetic Biology has seen the creation of certain circuits in this domain; from reconfigurable logic gate systems [23, 27] [28] to systems with switchable dynamical behavior [27, 29, 30]. Works like [29, 30] that propose a novel synthetic circuit, which can act both as an oscillator and a toggle switch have been a major influence on our work. Inspired from reconfigurable computer architecture that has been done by processing with very flexible high speed computing fabrics like field programmable gate arrays (FPGAs), genetic reconfigurable circuits could provide the solution to the limitation of using multiple circuits consisting of non-orthogonal parts, and also reduce the size of circuits, enabling multiple functionality from a single reconfigurable circuit.

Synthetic biology is at the intersection of biology, engineering and computational mathematics. Consequently, there are two distinct aspects to the design, analysis and implementation of a synthetic circuit: in-silico and in-vivo validation. Under the in-silico paradigm, we forego the specific implementation details, and focus on the abstract mathematical model and the dynamical behavior such a model allows. While for in-vivo analysis, we work on identifying the specific biological components that can be used to implement the identified topology for the synthetic network. Here, in order to create reconfigurable circuits in bacteria (*E*.*coli*), we ran simulations and provided a framework and proposed a circuit for the creation of reconfigurable networks that allow for switching of behavior dependent on the input given to the cells. We use optogenetics to implement the required behavior. Thus, the system implements an oscillator with a light dependent switch, which can work in one of two ways. The native system is a synchronized two component oscillator, which, dependent on the frequency of light shone on it, can be switched to a system which shuts off the oscillations completely, sending the system to a state of constitutive expression of A and no expression of B (A and B being the two components of the oscillator), or can create a system in which the frequency of the oscillations changes. This module is titled the ‘Highly Optogenetically Tunable Frequency Modulator (HOTFM)’. Oscillators can be used as clock references, and thus, controlling the frequency of oscillation would offer certain advantages. For instance, increasing the frequency might allow faster execution of a process and vice versa. In this regard, one possible medical application could be time-controlled drug delivery. If a drug is known to function optimally if delivered at distinct regular intervals, an oscillator handling the delivery of the drug would be an attractive option. Further, if under circumstances quite stark from the ordinary, the rate of delivery needs to be increased (decreased), our approach could be a viable option. We require two additions to an oscillator to realize HOTFM. A mechanism to modulate the frequency of the oscillations and an optogenetic module to act as a switch for this mechanism.

We lay the computational framework here for the creation of such circuits, and its scale up, which would allow switching to an even wider range of responses in complex systems.

## Materials and methods

### Danino oscillator architecture

**Fig 1:**
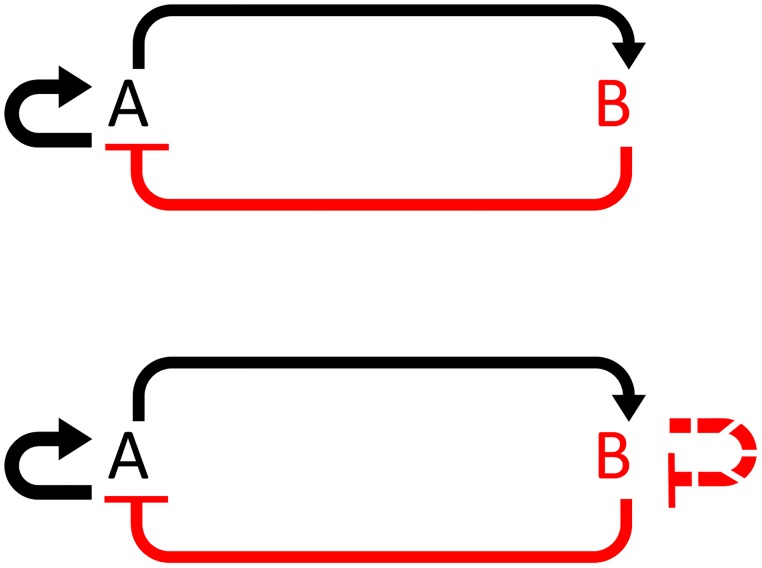
Topology of the Danino oscillator and HOTFM. (a) Abstract topology of the Danino oscillator. A (LuxI) activates itself and B (AiiA), whereas B (AiiA) represses A. (b) Abstract topology of the HOTFM. The topology remains largely the same, except that B undergoes negative autoregulation via self repression. It is suggested that here that this self-repression be mediated by optogenetics, using two component light response regulation systems such as [31, 32].

The Danino oscillator [23] is a two component oscillator consisting of an activator and a repressor, LuxI and AiiA respectively, Fig 1. LuxI produces small molecules called AHL which bind to the LuxR protein to form a transcriptional activator complex. This complex activates LuxI and AiiA, both of which are placed downstream of the Lux promoter. Whereas, AiiA encodes a lactonase which degrades the AHL molecules, thereby leading to an indirect repression of LuxI. The novelty of the Danino oscillator lies in its ability to generate synchronized oscillations in a colony of cells. This happens via AHL’s capability to diffuse across the cell membrane and be exchanged between cells akin to a communication signal for synchronizing the oscillators in each cell.

**Fig 2:**
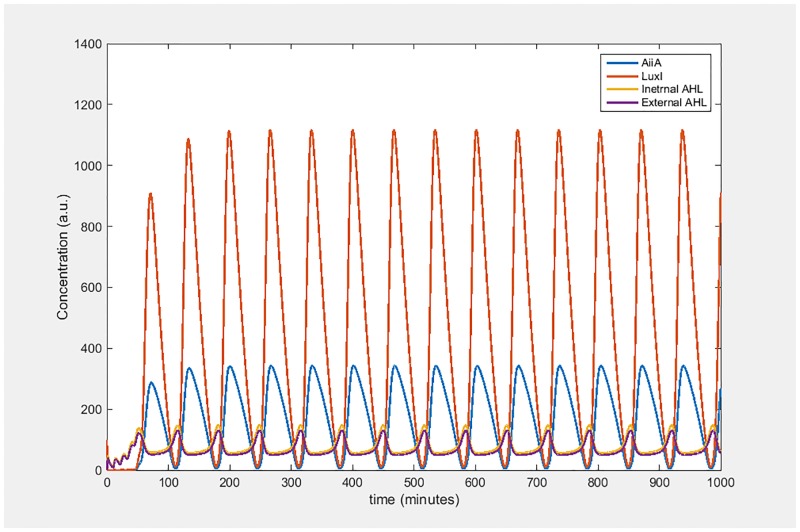
Dynamics of the Danino oscillator. Concentration versus time plots for the levels of AiiA, LuxI, internal AHL and external AHL.

The oscillations in the Danino oscillator can be divided into three phases: AHL buildup, AHL degradation and the resting phases. These phases can be seen in Fig 2. During the buildup phase AHL molecules accumulate in the system, and once the concentration reaches sufficient levels, there is a burst in the concentrations of both LuxI and AiiA. Concurrently, the increased AiiA values begin to degrade the accumulated AHL molecules. After the AHL levels have been brought down to a resting state, both LuxI and AiiA concentrations start to fall, owing to enzymatic degradation; following which we again have the buildup phase. In this manner the oscillations continue.

### Danino oscillator: Mathematical model

The Danino oscillator is modelled using a set of delay differential equations consisiting of four equations, one for each AiiA, LuxI, internal AHL and external AHL concentrations [23]. We recapitulate the model for the Danino oscillator here from Eqs 1–4. This is a deterministic model for intracellular concentrations of LuxI(*I*), AiiA(*A*), internal AHL(*H_i_*) and external AHL(*H_e_*)

$$\begin{array}{c} \frac{\partial A}{\partial t}=C_{A}\left[1-{\left(\frac{d}{d_{0}}\right)}^{4}\right]P\left(\alpha ,\tau \right)-\left(\frac{\gamma_{A}A}{1+f\left(A+I\right)}\right) \end{array}$$ (1)

$$\begin{array}{c} \frac{\partial I}{\partial t}=C_{I}\left[1-{\left(\frac{d}{d_{0}}\right)}^{4}\right]P\left(\alpha ,\tau \right)-\left(\frac{\gamma_{I}I}{1+f\left(A+I\right)}\right) \end{array}$$ (2)

$$\begin{array}{c} \frac{\partial H_{i}}{\partial t}=(\frac{bI}{1+kI})-(\frac{\gamma_{H}AH_{i}}{1+gA})+D\left(H_{e}-H_{i}\right) \end{array}$$ (3)

$$\begin{array}{c} \frac{\partial H_{e}}{\partial t}=-(\frac{d}{1-d})D\left(H_{e}-H_{i}\right)-\mu H_{e}+D_{1}\frac{\partial^{2}H_{e}}{\partial t^{2}} \end{array}$$ (4)

In Eqs 1 and 2 the term

$$\begin{array}{c} P\left(\alpha ,\tau \right)=\frac{\delta +\alpha H_{\tau }^{2}}{1+k_{1}H_{\tau }^{2}} \end{array}$$

accounts for the time-delayed effect of AHL on AiiA and LuxI through the concentration of the internal AHL *H_τ_(t)* = *H_i_(t-τ)*. Description for the various parameters in Eqs 1–4 can be found in [23]. A summary of the parameters is given as follows:

1. *A* = Concentration of AiiA
2. *I* = Concentration of LuxI
3. *H*_i_ = Concentration of internal AHL
4. *H*_e_ = Concentration of external AHL
5. *γ_A_* = Enzymatic degradation of AiiA; *γ_I_* = Enzymatic degradation of LuxI; *γ_H_* = Enzymatic degradation of internal AHL
6. *d* = Cell Density
7. *D* = Diffusion of AHL through cell membrane
8. *D_1_* = AHL Diffusion Rate
9. *μ* = external AHL decay rate (flow rate)
10. *τ* = Time delay
11. *f*, *g*, *b*, *k* = Michaelis-Menten kinetics parameters for enzymatic degradation of proteins
12. *δ* = Basal Rate
13. *α*, *k_1_* = Hill Function Coefficients
14. *d* = Cell Density

Eqs 1–4 quantitatively capture the qualitative picture presented in Danino Oscillator Architecture. Specifically, the last term in Eq 4 captures the synchronized nature of the oscillators; the movement of AHL across the cell membranes allows the cells to communicate with their neighbours regarding the state of oscillations.

### HOTFM: Architecture

The Danino oscillator has a linear relationship between amplitude of oscillations and the time period [23], owing to the fact that the time period is dictated by the time it takes for the AiiA and LuxI concentrations to degrade. One possible way to bring down the time period of oscillations would be to reduce the length of this degradation period. Thus, we could, in principle, modulate the amplitude to modulate the frequency of oscillation. We propose to do this by adding negative autoregulation on AiiA Fig 1. This transforms the amplified negative feedback oscillator topology of the Danino oscillator into a smolen oscillator [19]. The Smolen oscillator is known to have scope for tunability of the frequency response [19, 33].

To control the negative regulation of AiiA we need a reliable way to switch on and off this pathway. For this we use optogentics as a flexible and easy mechanism. Optogenetics is a method that is quickly gaining traction for regulation of gene expression. This is due to the fact that such systems offer dynamic switching between the active and inactive state (light systems typically switch from ON to OFF state in the order of seconds to minutes) [34], and the input can be varied instantaneously by varying the intensity of light as opposed to chemical systems where a difference in chemical inducer concentration requires time to be diffused into the system and achieve a uniform concentration gradient. Thus, optogenetic systems have found applications in several fields such as in silico feedback [35, 36] and control [31, 37], understanding cell signaling pathways [38] and neuroscience [39] The self-repression pathway in our proposed system thus, is put under the control of a repressor, such as TetR; and this repressor is controlled by a two component light response system [31], which is an optogenetic module. So, whenever the oscillator is required to change its frequency of operation, we can, in principle, shine light on the cells and the self-repression pathway (mediated by the repressor) would be activated. Further, in order to ensure that the repression is a self-repression, i.e. in proportion to the amount of AiiA present, we have the two genes of the two component system placed under the same controls as that of AiiA, with the promoter and degradation tag used for both AiiA and the two component system being the same.

### HOTFM: Mathematical modelling

The model for HOTFM is based on that of the Danino oscillator, Eqs 1–4 [23]. To incorporate optogenetically triggered negative autoregulation on AiiA, we add the following two equations to the set of differential equations for the Danino oscillator:

$$\begin{array}{c} \frac{\partial R}{\partial t}=\left[1-{\left(\frac{d}{d_{0}}\right)}^{4}\right]\left(\frac{\alpha_{TC}TC^{n_{TC}}}{TC^{n_{TC}}+K_{TC}^{n_{TC}}}\right)-\left(\frac{\gamma R}{1+fR}\right) \end{array}$$ (5)

$$\begin{array}{c} \frac{\partial TC}{\partial t}=C_{A}\left[1-{\left(\frac{d}{d_{0}}\right)}^{4}\right]P\left(\alpha ,\tau \right)\left(\frac{K^{n_{1}}}{R^{n_{1}}+K^{n_{1}}}\right)-\left(\frac{\gamma_{A}TC}{1+f\left(TC+I\right)}\right) \end{array}$$ (6)

and modify Eq 1,

$$\begin{array}{c} \frac{\partial A}{\partial t}=C_{A}\left[1-{\left(\frac{d}{d_{0}}\right)}^{4}\right]P\left(\alpha ,\tau \right)\left(\frac{K^{n_{1}}}{R^{n_{1}}+K^{n_{1}}}\right)-\left(\frac{\gamma_{A}A}{1+f\left(A+I\right)}\right) \end{array}$$ (7)

where,

1. *R* = concentration of the repressor;
2. *TC* = concentration of TC regulator
3. *α*_*CI*_ = Hill function coefficient of CI repressor
4. *K* = Repression coefficient for the repressor
5. *K*_*TC*_ = Activation coefficient for TC
6. *n*_*R*_ = cooperativity of the repressor
7. *n*_TC_ = cooperativity of TC
8. *γ* = degradation coefficient for the repressor
9. *γ*_*TC*_ = degradation coefficient for TC

Eq 5 quantifies the kinetics of the repressor, which is activated by the two component light system via hill function type activation. Akin to the Danino oscillator, the prefactor *[1-(d/d_0_)^4^]* accounts for the limitation in the protein production owing to nutrient limitation and waste accumulation [23]. Both the two component system and AiiA are repressed by the repressor Eqs 6 and 7 via hill type repression.

### HOTFM: Proposed biological implementation

**Fig 3:**
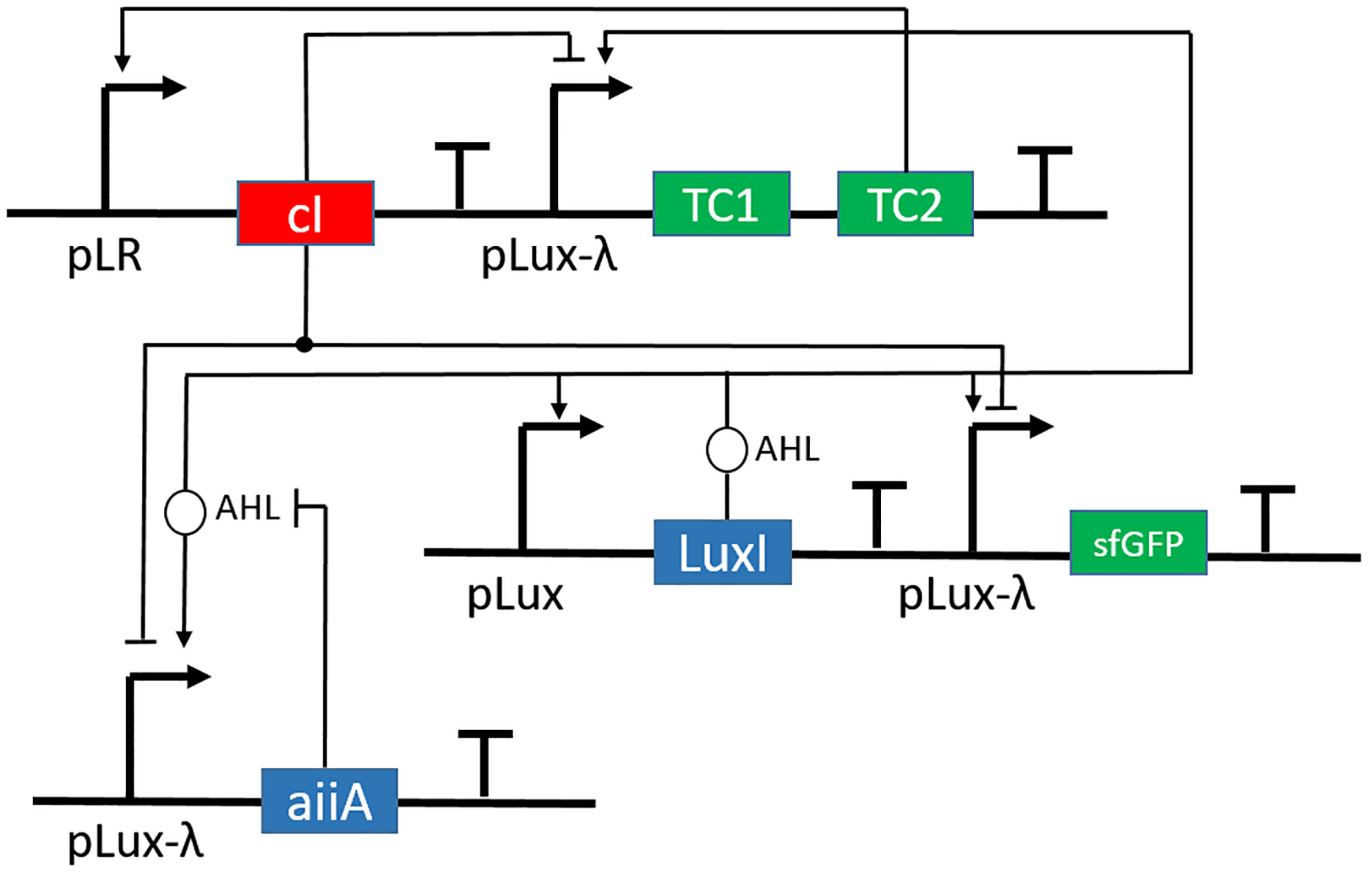
Circuit diagram for HOTFM. In the biological implementation of the circuit, the LuxI is placed under the Lux promoter, similar to that in the Danino oscillator. The AiiA gene, however, is placed under a Lux-Tet promoter which can be activated by AHLs produced by LuxI and repressed by the lambda repressor. TC1 and TC2 represent the two genes of the two component light response systems, CcaS-CcaR and UirS-UirR. pLR denotes the light response promoter. Thus, in the actual circuit, there would be two copies of the TetR repressor, one under pLR1 (pCPCG2) and the other under pLR2 (pcsiR1). The output has been connected across AiiA via sfGFP under the same controls as AiiA. The hammerheads represent repression while the arrows represent activation of the particular promoter where the hammerheads and arrows end.

The core of the HOTFM consists of an architecture similar to the Danino oscillator [23], with the two components being LuxI (A) and AiiA (B), that function in the same manner as described in Danino Oscillator Architecture. In this system, however, the LuxI gene is under the Lux promoter, while the AiiA gene is under a hybrid Lux-Tet promoter, which is activated by AHL but repressed by a repressor such as TetR. This modification has been added in order to facilitate an auto self-repression step on the AiiA component. To achieve this, we propose to express two two component light response systems, namely the CcaS-CcaR and the UirS-UirR systems [31, 32], both under the control of hybrid Lux-Tet promoters, in order to ensure that at any point in time, the level of expression of these light response systems is identical to the expression of AiiA, which is crucial for self-repression. Additionally, it may be noted, that these two systems are orthogonal to each other as the wavelength of light for activation for the two systems are different, with the CcaS-CcaR system being activated under green light and deactivated under red light, while the UirS-UirR is activated under UV-violet light and also turned off under green light. In this cascade, we also express two copies of a repressor, such as TetR, one under the CPCG2 promoter driven by phosphorylated CcaR and the other under the csiR1 promoter, driven by phosphorylated UirR. Oscillations are monitored by a sfGFP reporter, connected downstream of a hybrid Lux-Tet promoter, which measures AiiA levels. The circuit diagram is shown in Fig 3. In the figure, TC1 and TC2 represent the genes of the two component light response systems (TC), of which TC2 ultimately acts as the activator. The other symbols have their usual meaning.

The proposed system then, would work as follows—In the native dark state, when no light is being shone on the system, the cells would have a behavior similar to that described by [23], with a burst of transcription leading to a buildup of luxI, AHL and AiiA, which would quench the AHL and bring the system to its native state and repeat, driving the oscillations. In our system, the same behavior occurs, but along with this, the CcaS, CcaR, UirS and UirR proteins are being produced. These proteins however, are in an inactivated state, due to a lack of activating light. From here, our system can be switched into one of two states. If green light of wavelength 520nm is shone upon the system, it drives a conformational change in the membrane bound sensory kinase CcaS, which then phosphorylates the response regulator CcaR, which can now activate transcription of genes placed under the CPCG2 promoter. This CcaR therefore, activates the production of the repressor, which represses the production of aiiA transcriptionally. This regulation, coupled with AHL degradation that is catalyzed by AiiA bring down the levels of AHL faster than the native oscillator (without the light response system), thereby not allowing the oscillations to go up as high as the former case, bringing down the mean amplitude and consequentially increasing the frequency of the oscillations. This behavior is in accordance with the simulations by [23], which showed that the oscillator had a linear relationship between amplitude and time period. The other state of the HOTFM can be driven by shining UV-Violet light of wavelength 405 nm, which would activate the UirS-UirR system in a similar manner as the CcaS-CcaR system, leading to activation of the repressor under the csiR1 promoter. However, since the dynamics of the UirS-UirR system are different from that of the CcaS-CcaR system, the same repressor would have a different effect on the oscillations Results and Discussions. In this case, due to a shift in the dynamics of the light response system, the repressor has a higher activation under the same intensity of light, and thus represses all the AiiA, leading to a stop in the oscillations. This sends AiiA to a constitutive OFF state, while the LuxI now is free of repression and can self-amplify itself and go to a constitutive ON state, thus killing the oscillations.

### Synchronization

Synchronization is one of the properties central to the Danino oscillator [23], as it presented the first biological oscillatory circuit capable of synchronizing over a small colony of around 200 cells. Previously developed oscillators [21, 33] had single cells oscillating in either mircrofluidic chambers or tailored environments without synchronization. There are many concepts of synchronization, such as amplitude and frequency synchronization. The Danino oscillator presented a case of phase synchronization, wherein the oscillations among different cells, are present initially in different phases (due to stochasticity). Over time, the oscillators synchronize, due to coupling of oscillators, when they align to a common phase in their oscillations spontaneously [40]. This general definition of phase synchronization holds true across all domains, and has applications over vast areas of physics, biology and other engineering fields. The system discussed in this paper in particular, the Danino oscillator, from which the HOTFM architecture has been inspired, this coupling is achieved via a quorum sensing molecule called AHL. The AHL, produced in the system due to the presence of the LuxI gene in the circuit, diffuses across the cell membrane, entering neighboring cells, leading to coupling. This diffusion of AHL appears in the model as a partial second order derivative in the equation for external AHL, governed by Fick’s second law of diffusion. This is a unique feature bestowed upon the cells only due to the oscillator circuit being added to them, as the *E*.*coli* cells naturally do not produce AHL or any molecule which could act as a means to couple the cells. When the oscillator circuit is added to them, AHL is produced due to the transcription and translation of the LuxI gene which is critical for oscillations, and produces AHL leading to coupling. This circuit, thus, when transformed into any cell would cause AHL generation and coupling, which is the key factor for synchronization.

### Robustness

Robustness is an important concern in synthetic genetic circuits. As the field of synthetic biology grows, the number of circuits being proposed for a variety of applications is rising exponentially. However, a major difficulty associated with the development of new circuits is that they do not function as expected due to uncertain initial conditions and disturbances in the environment, which can cause the host cell to not produce the desired response [41]. Analysis of robustness, thus, is essential for the design of synthetic circuits [42–44]. There are several definitions of robustness, presented with different aspects in mind [45–47]. The basic idea behind robustness is the property of a system to be resistant and maintain its state against perturbations [48]. Here, we stick to a narrow definition of robustness, ie transient robustness. In order to achieve this, we study the regions in the parameter space of degradation rate of LuxI and AiiA, and also LuxI and AHL, in which oscillations are obtained for both the Danino oscillator and the HOTFM. In this region we observe the rate of synchronization in the two oscillations, and note down which oscillator synchronizes faster and is therefore more transiently robust over the entire parameter space. Additionaly, we look at the integrated absolute synchronization error (IASE) for quantifying the extent of deviation from synchronization; The oscillator with a lower IASE would be more robust Synchronization: HOTFM is more Robust compared to the Danino Oscillator.

## Results and discussions

### Assessment of the proposed synthetic circuits

The two main hypotheses of this work are concerned with tunability of frequency of oscillations and robustness of synchronization. The former is a property at the cell level whereas the latter concerns the collective behavior of a colony of cells. Thus, we analyze the system in both these scenarios.

#### Single cell study

To conduct insilico assessment of the proposed synthetic circuit ‘bulk’ simulations are performed by dropping the diffusion term *D_1_* in Eq 4 as suggested in [23]. The resulting dynamical model for HOTFM Eqs 2–4, 5–7 is simulated with respect to the parameter of the repressor hill function *K* introduced in Eq 7 and the degradation coefficient for the repressor *γ* introduced in Eq 5. Numerical Integration of a system of differential equations is sensitive to the initial conditions assumed for the involved variables; and only converge to the stable solutions. Thus, it is not conducive for eliciting the qualitative dependence of the solution on the parameters of the system. In this work, numerical bifurcation analysis has been employed for this purpose; the change in behavior of the HOTFM oscillator is tracked in the two parameter space of *K* and *γ* using numerical bifurcation performed with the Matlab package DDE-BIFTOOL [49]. Further, numerical intergation is performed with the Matlab solver dde23 for obtaining representative solutions in the different regions identified via numerical bifurcaton.

#### Colony level study

Synchronization is a population level behavior, thus for studying robustness of synchronization a linear colony of 6 cells is assumed for both HOTFM and the Danino oscillators. For each cell in a colony a dynamical model is formulated using Eqs 1–4 for the Danino oscillator and Eqs 2–4, 5–7 for HOTFM. The diffusion term *D_1_* in Eq 4 is retained for this experiment since this parameter accounts for the coupling between the cells in a colony. Two two-parameter numerical bifurcations are conducted for both HOTFM and the Danino oscillators. The parameter spaces for this purpose are *γ_I_*-*γ_A_* and *γ_H_*-*γ_A_*; where, *γ_A_*, *γ_I_* and *γ_H_* represent the enzymatic degradation for AiiA, LuxI and internal AHL respectively. For each parameter space, transient synchronization behavior is studied in the region of parameters where oscillations are feasible; further, for a comparative analyis, the region of intersection for HOTFM and the Danino oscillators is considered.

A narrow view of robustness, limited to transient behavior of synchronization, has been adopted in this work Robustness. To quantify this behavior two metrics to judge the level of synchronization in a colony of cells is used. These metrics are defined as follows:

1. Integrated Absolute Synchronization Error (IASE)—We define Absolute Synchronization error (ASE) as the difference in the concentrations of AiiA (sfGFP reporter measures AiiA levels) between neighbouring cells, normalized by the amplitude of oscillations and averaged across all the cells. Consider a linear colony of n cells, either Danino or HOTFM. At time *t = 0* the levels of LuxI are set to 100 arbitrary units (a.u.) and additive uniform noise is added from the interval 0–400 a.u.; All the other concentrations are set to zero at time *t = 0*. We also apply periodic boundary conditions. The n systems of differential equations are numerically integrated for 500 minutes. We define ASE as
  $$\begin{array}{c} ASE\left(t\right)=(\frac{(\sum_{i=1}^{n-1}|A_{i}\left(t\right)-A_{i+1}\left(t\right)|)+|A_{n}\left(t\right)-A_{1}\left(t\right)|}{n}) \end{array}$$ (8)
  Where *A_i_* represents the concentration of AiiA at the *i*th cell at time t. ASE captures the absolute deviation of the colony of cells from synchronization at time *t*. To calculate an estimate of overall deviation we integrate ASE over the time span of numerical intergration *t_final_*, which is 500 minutes in this case, to give Integrated Absolute Sychronization Error (IASE).
  $$\begin{array}{c} IASE=\int_{0}^{t_{final}}ASE\left(t\right)dt \end{array}$$ (9)
  The IASE values are compared between HOTFM and the Danino oscillators for ascertaining the robustness behavior for synchronization. The difference *IASE_Danino_ − IASE_HOTFM_* is used as the comparative metric; a positive (negative) value indicating greater (lesser) robustness for HOTFM compared to the Danino oscillator.
2. Rate of Synchronizaton (r)—In addition to total deviation from synchronization, we also measure the rate at which the effect of noise introduced at time t = 0 on synchronization dies down. Equivalently, this rate gives the rate of synchronization (r). We fit an exponential Envelope to the ASE (EASE) and use the exponent of this fitted exponential envelope as an estimate of r.

$$\begin{array}{c} EASE=a*exp(-r*t) \end{array}$$ (10)

The r values are compared between HOTFM and the Danino oscillators for ascertaining the robustness behavior for synchronization as well. The difference *r_HOTFM_ − r_Danino_* is used as the comparative metric; a positive (negative) value indicating greater (lesser) robustness for HOTFM compared to the Danino oscillator.

**Fig 4:**
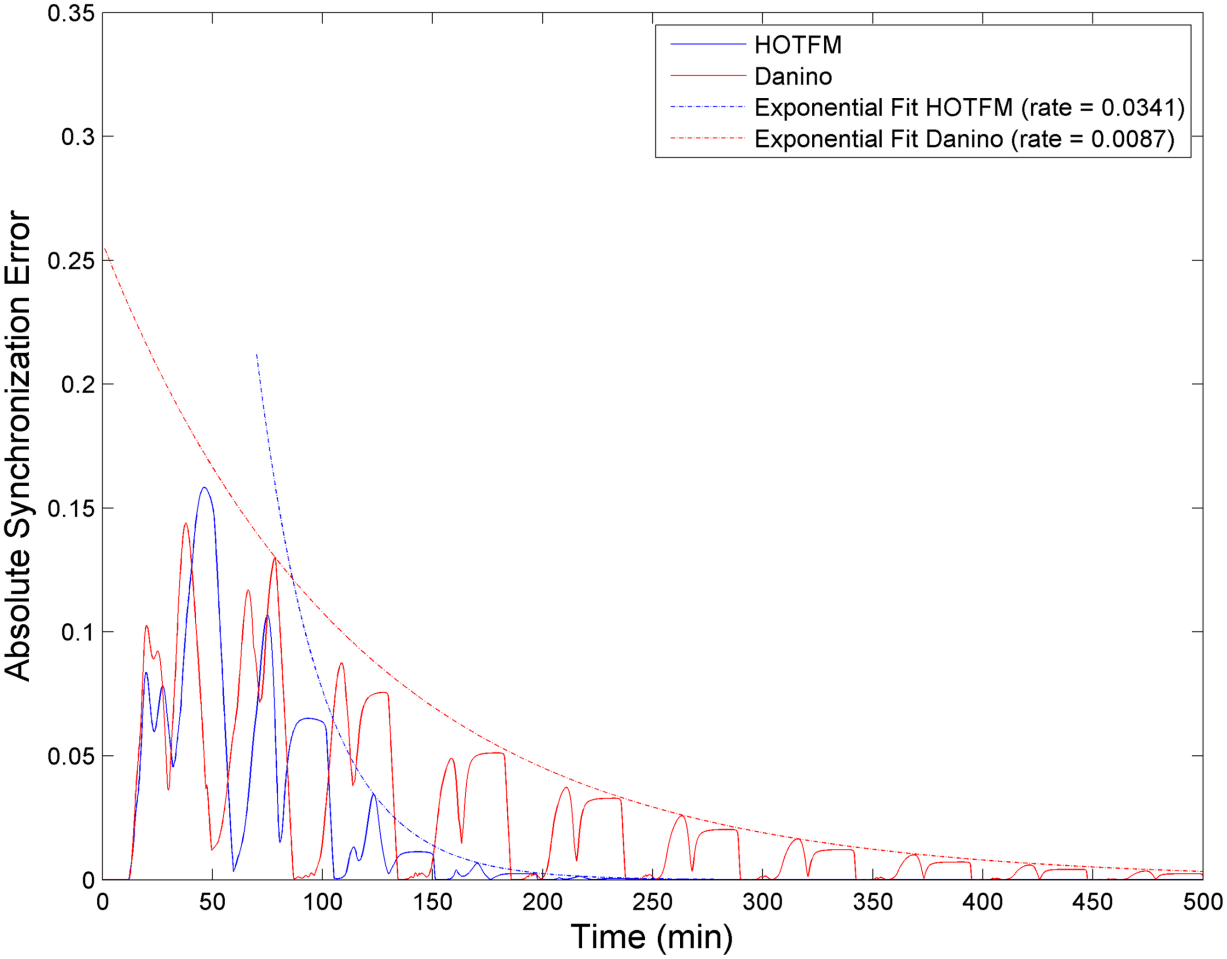
Absolute Synchronization Error (ASE) and Rate of Synchronization. Plots for the Absolute Synchronization Error (ASE) and Rate of Synchronization (r) for HOTFM and the Danino Oscillators. Solid curves represent ASE (Red curve for Danino and Blue curve for HOTFM). The dotted curves are obtained after fitting an exponential envelope of the form *EASE* = a*exp(−r*t) to the ASEs (Red curve for Danino and Blue curve for HOTFM). The values for the parameters are adapted from [23], except for *γ_A_* and *γ_H_*; *γ_A_ = 23* and *γ_H_ = 0.023*. For the equations unique to HOTFM, Eqs 5 and 6, the hill function parameters for the two component system are adapted from [31] for CcaS-CcaR. TetR is used as the repressor downstream of Ccas-CcaR with a repression coefficient *K* = *100* and degradation constant *γ* = *1.5*.

Fig 4 shows a representative example of ASE and r for HOTFM and the Danino oscillators. In these experiments the values for the parameters are adapted from [23], except for the free parameters. For the equations unique to HOTFM, Eqs 5 and 6, the hill function parameters for the two component system are adapted from [31] and [32] for CcaS-CcaR and UirS-UirR, respectively. For the study of robustness of synchronization the diffusion constant *D_1_* is set to a value of 100 *μm^2^*/*s*.

The Matlab scripts for the bifurcation and time domain analyses have been provided as supporting information in S1 Code.

### Bifurcation analysis reveals Hopf bifurcation for HOTFM

**Fig 5:**
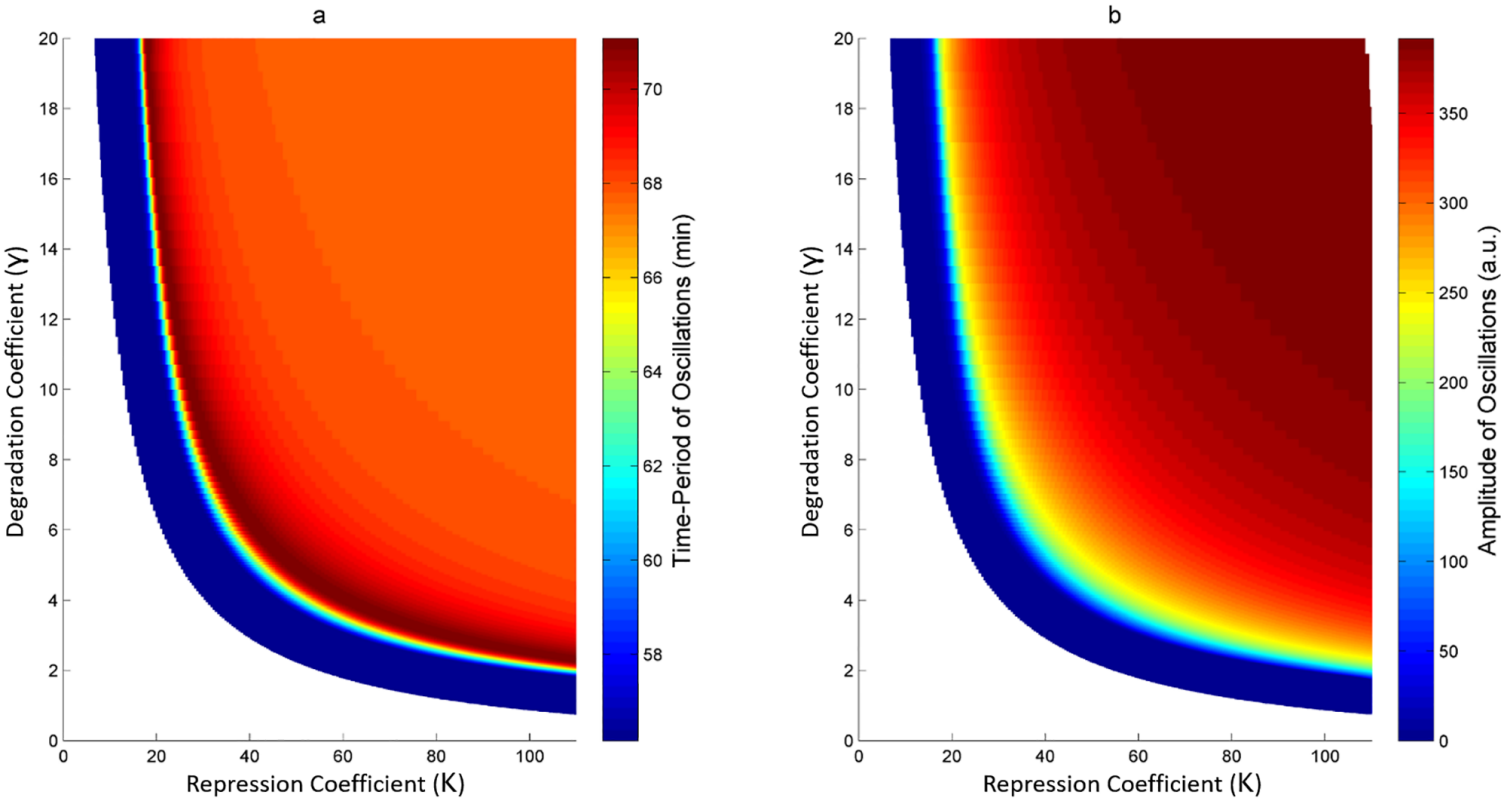
Two parameter bifurcation for HOTFM in the repression coefficient (*K*)-degradation cefficient for repressor (*γ*) space. Two parameter bifurcation for HOTFM against the parameters for the repressor downstream of the two component light system (CcaS-CcaR); the x-axis corresponds to the repression coefficient *K* and y-axis corresponds to the degradation coefficient *γ*. The boundary of the shaded region represents the branch of hopf solutions; the unshaded region below the hopf branch corresponds to parameter values for which there exits a stable steady state and oscillations are absent. Whereas, the shaded region above the hopf branch represents parameter values for which stable oscillations exist. a) The two parameter bifurcation plot with the shaded region representing a heatmap for the period of oscillation. At each point in this region, the color represents the period of oscillation. Points farther away from the hopf branch in the shaded region show higher period (lower frequency), while the points closer to the hopf branch exhibit lower period (higher frequency). b) The two parameter bifurcation plot with the shaded region representing a heatmap for the period of oscillation. At each point in this region, the color represents the period of oscillation. Points farther away from the hopf branch in the shaded region show higher amplitude, while the points closer to the hopf branch exhibit lower amplitude.

Fig 5 shows the two-parameter bifurcation for HOTFM. We see two qualitatively different stable behaviors here—stable steady state and stable oscillations, separated by a branch of hopf solutions. The region below the hopf branch corresponds to the parameter values for which there exists a stable steady state; and the region above the hopf branch contains region for oscillatory behavior. It is evident that the branch of hopf solutions follows a hyperoblic trend: as the strength of the repression coefficient increases for the repressor, the degradation rate for which the system transitions from a stable steady state solution to a stable oscillatory solution decreases. Low value for the repression coefficient suggests a strong repressor, which has the potential to kill the oscillations at the AiiA node. Intuitively, this behavior can be counteracted by employing strong degradation tags that afford high degradation rates [50], thus allowing the oscillations to continue. Conversely, for weak repressors the repression coefficients take on large values, thus even weak degradation tags would suffice to ensure sustenance of oscillations; for weak repressors the system becomes equivalent to the Danino oscillator since the hill function component due to the repressor tends to unity for large values of the repression coefficient. The bifurcation plots of Fig 5 can be used to select the feasible values for the degradation tag for which a given repressor, such as TetR, would kill the oscillations.

### HOTFM modulates the frequency and amplitude of oscillations

**Fig 6:**
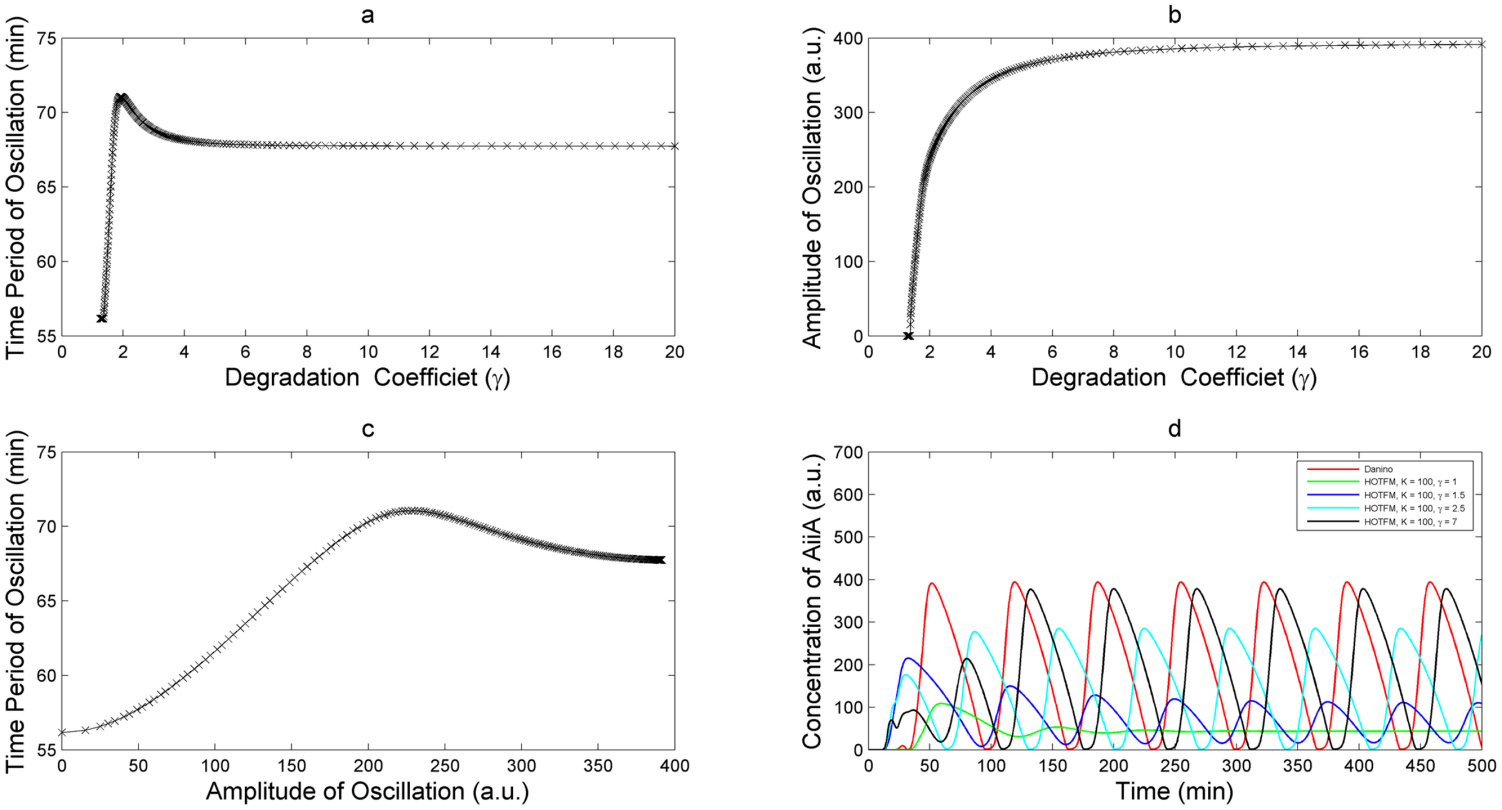
Frequency and amplitude variation for HOTFM implemented with TetR repressor. Variation in the frequency and amplitude of oscillation for AiiA with changing value of the degradation coefficient *γ* for HOTFM when TetR is used as the repressor downstream of the two component CcaS-CcaR. The repression coefficient *K* assumes a value of 100 for TetR [51]. At low values of the degradation coefficient (below the hopf branch) oscillations die and there is presence of a stable steady state. However, as the degradation rate increases and the hopf branch is crossed both amplitude and time period increase with increasing degradation coefficient. On the other hand, the period overshoots before settling to values equivalent to the Danino oscillator. Time period and amplitude have a largely linear relationship with increasing *γ*. a) Variation in time period with increase in degradation coefficient *γ* for AiiA. b) Variation in amplitude of oscillation with increase in degradation coefficient *γ* for AiiA. c) Time period vs amplitude as the degradation coefficient *γ* is increased. d) Numerically integrated solution for HOTFM at different values of the degradation coefficient *γ*.

As hypothesized in HOTFM: Architecture, simulations confirmed the modulatory effect of adding self repression on AiiA. In regions farther away from the hopf branch within the oscillatory region, both the period and amplitude of oscillations increases Fig 6, finally tending to behaviors equivalent to the Danino oscillator. However, the period overshoots above the Danino oscillator for a while, and finally settles into the Danino oscillator; this behavior is not present in the amplitude heatmap. For illustrative purposes, we select the TetR repressor for which the repression coefficient *K* is 100, after suitable scaling [51].

**Fig 7:**
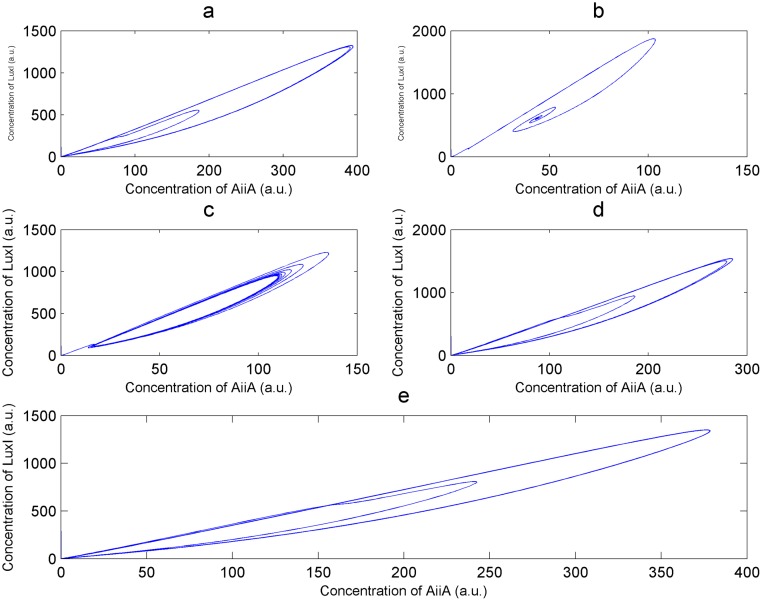
Phase plane plots. Phase plane plots for the different numerically integrated solutions shown in Fig 6d. AiiA concentration is plotted on the x-axis while LuxI concentration is plotted on the y-axis. Equivalent to the results from the time series plots, the phase plane plots show the variation in behavior with increasing degradation coefficient *γ*.

A strategy for tuning the response of the Danino oscillator using self-repression can be suggested from Fig 6a–6c. For a given repressor, this can be achieved by varying the strength of the degradation tag. A low value for the degradation tag would temporarily kill the oscillations when appropriate light is shone on the system; such an arrangement can be employed to design an optogenetically controlled kill switch for oscillations. Once the hopf branch is crossed, oscillations survive for all values of the degrdation tag henceforth. Within this region amplitude can be traded-off for higher frequeny of oscillation (lower period of oscillation). Staying closer to the hopf branch would afford highest frequencies of operation at the expense of lowering the amplitude. This can equivalently be seen in Fig 6d where numerically integrated time series solutions are depicted from different regions of the bifurcation diagram. These time-domain plots are plotted for the TetR repressor, for which a value of 100 was used for the repression coeffcient (reference here) after appropriate scaling. Fig 7 depicts the phase plane plots corresponding to the time series shown in Fig 6d.

Self-repression on AiiA as a means to tune the frequency response was conjectured from the linear relationship between the period and amplitude of oscillatons for the Danino oscillator [23]. The heatmaps in Fig 5 confirm positive correlation between the period and amplitude of oscillation for HOTFM. Fig 6c shows the variaton of the period and amplitude of oscillation along the branch of periodic solutions emanating from the hopf branch for the TetR repressor as the strength of the degradation tag is varied. Fig 6a and 6b again depict the increasing behavior of the period and amplitude of oscillations with increase in strength of the degradation tag. The linear relationship between the period and amplitude of oscillation is confirmed from Fig 6c for the TetR repressor.

A greater reconfigurability can be achieved by combining both the kill switch and frequency modulating behavior into a single circuit. As discussed in HOTFM: Proposed Biological Implementation, we can use both the CcaS-CcaR and the UirS-UirR systems in a single configuration by placing two different copies of the TetR repressor downstream to the CPCG2 and csiR1 promoters respectively. The repression coefficient of TetR repressor is around 100 [50]. The dynamics of the UirS-UirR systems is qualitatively similar to the Ccas-Ccar system [31, 32]. This suggests that similar to the Ccas-CcaR system we can modulate the frequeency response using a repressor with varying strength of the degradation tags. So, if we use a degradation rate of 1.5 with the TetR repressor associated to the CcaS-CcaR system and that with an appropriate value associated to the UirS-UirR, we can get a system that can exhibit three different types of response. In the absence of any light source, the system will oscillate like a normal Danino oscillator; in the presence of green light the Ccar-Ccas will get activated and as can be seen from Fig 6, the system will oscillate at a frequency higher than the Danino oscillator. If now the Ccas-Ccar system is switched off by shining red light on the system followed by Ultraviolet light, we see from (refer supplementary) that the oscillations will shut down.

### Synchronization: HOTFM is more robust compared to the Danino oscillator

**Fig 8:**
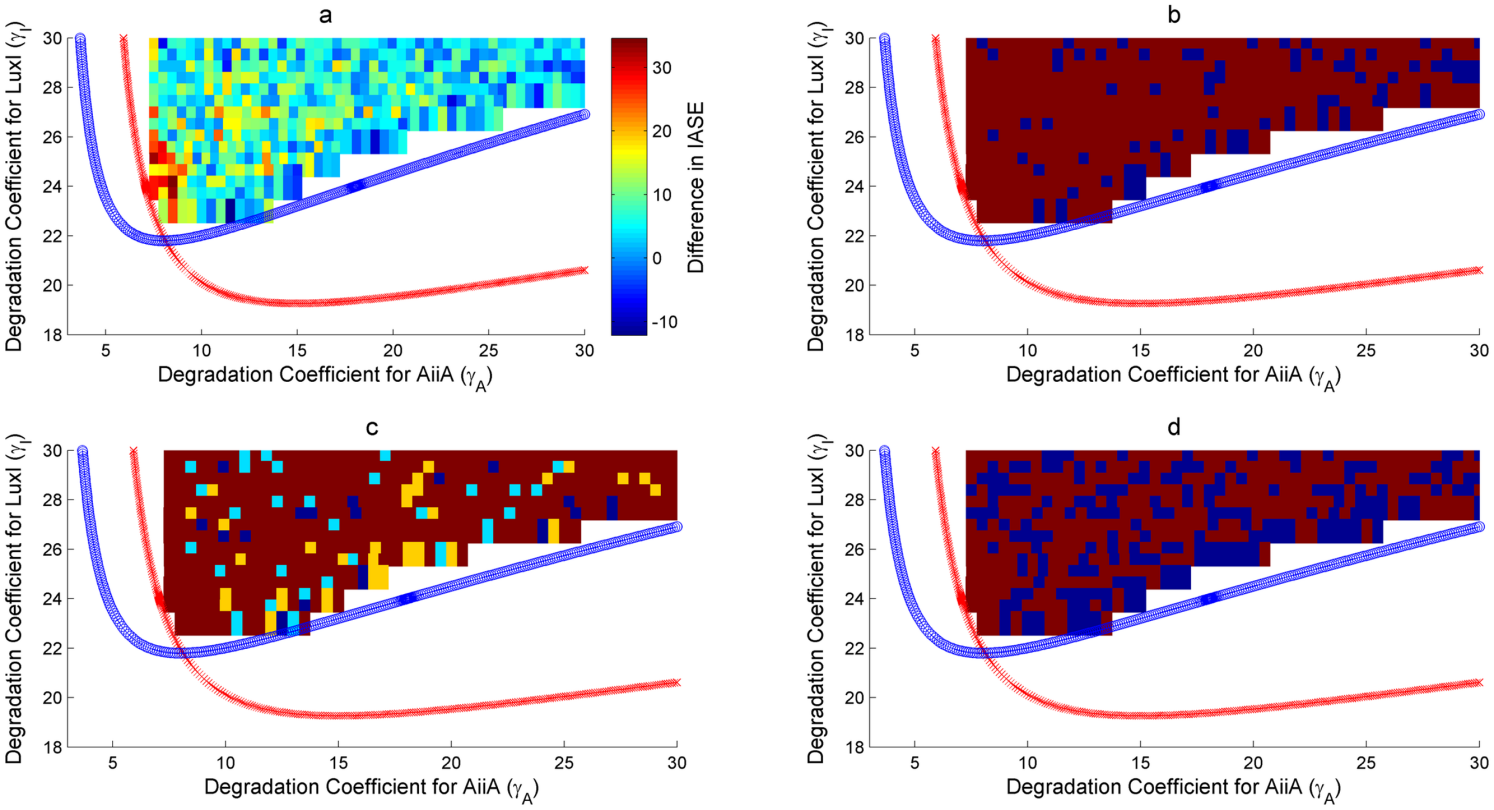
Robustness analysis for IASE in the *γ_I_*-*γ_A_* parameter space. Robustness analysis with respect to Integrated Absolute Synchronization Error (IASE). Two parameter bifurcation plots are presented for HOTFM and the Danino oscillators in the *γ_I_*-*γ_A_* parameter space for IASE. The blue (red) curve respresents the hopf branch for HOTFM (Danino). Region above the hopf branch corresponds to parameter values where oscillations occur, while for regions below the hopf branch oscillation dies and steady state solution arises. For a comparative analysis of robustness between HOTFM and Danino we consider 520 points in the region of oscillations common to both HOTFM and Danino. At each point the system of equations (containing a linear array of 6 cells for both HOTFM and Danino) is numerically integrated for 500 minutes. From the obtained solutions IASE is calculated. For points which have *IASE_Danino_* − *IASE_HOTFM_* > 0, HOTFM is more robust compared to Danino, while for *IASE_Danino_* − *IASE_HOTFM_* < 0 Danino is more robust. a) Heatmap representing *IASE_Danino_* − *IASE_HOTFM_* at the 520 points in the oscillatory region common between HOTFM and Danino. Positive values suggest that HOTFM is more robust for IASE compared to Danino and vice versa. b) Heatmap for part a. thresholded at the value 0. Regions with positive values in a. have dark red color here, while regions with negative values in a. are colored blue. c) Heatmap for the effect sizes for individual points calculated using bootstrap estimates for the distribution for *IASE_Danino_* − *IASE_HOTFM_* Robustness Analysis for IASE. If *IASE_Danino_* − *IASE_HOTFM_* > 0, effect size is reported for greater robustness of HOTFM, while if *IASE_Danino_* − *IASE_HOTFM_* < 0, effect size (cohen’s *d*) is reported for greater robustness of Danino. The color scheme is as follows—Blue (negligible effect, *d* < 0.2), Cyan (small effect, 0.2 ≤ *d* < 0.5), Yellow (medium effect, 0.5 ≤ *d* < 0.8) and Dark Red (large effect, d ≥ 0.8). d) Heatmap for the presence or absence of 0 in the 95% bootstrapped confidence interval. Dark red means absence of 0 while blue represents presence.

**Fig 9:**
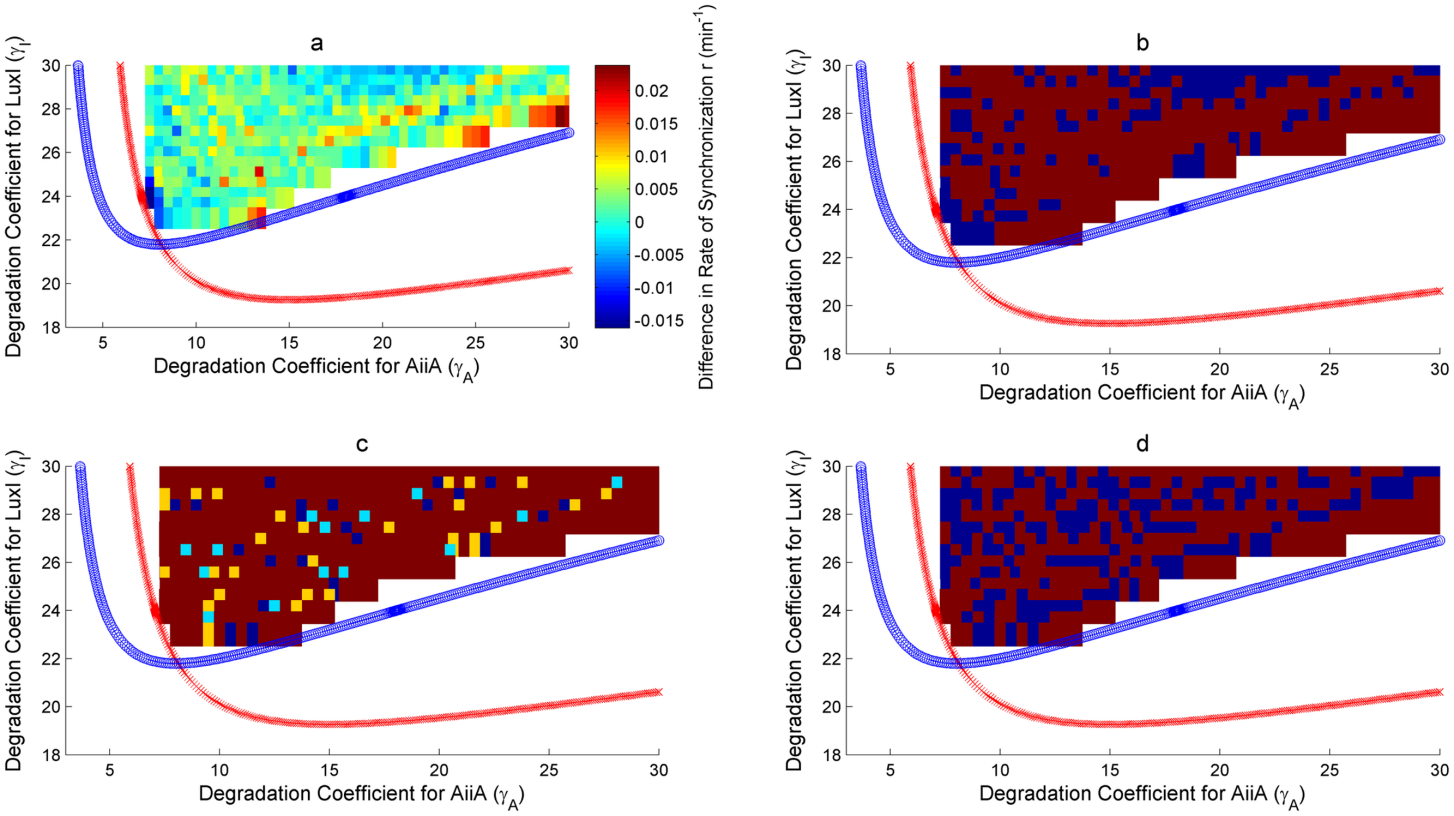
Robustness analysis for Rate of Synchronization (*r*) in the *γ_I_*-*γ_A_* parameter space. Robustness analysis with respect to Rate of Synchronization (*r*). Two parameter bifurcation plots are presented for HOTFM and the Danino oscillators in the *γ_I_*-*γ_A_* parameter space for *r*. The blue (red) curve respresents the hopf branch for HOTFM (Danino). Region above the hopf branch corresponds to parameter values where oscillations occur, while for regions below the hopf branch oscillation dies and steady state solution arises. For a comparative analysis of robustness between HOTFM and Danino we consider 520 points in the region of oscillations common to both HOTFM and Danino. At each point the system of equations (containing a linear array of 6 cells for both HOTFM and Danino) is numerically integrated for 500 minutes. From the obtained solutions *r* is calculated. For points which have *r_Danino_* − *r_HOTFM_* < 0, HOTFM is more robust compared to Danino, while for *r_Danino_* − *r_HOTFM_* > 0 Danino is more robust. a) Heatmap representing *r_Danino_* − *r_HOTFM_* at the 520 points in the oscillatory region common between HOTFM and Danino. Negative values suggest that HOTFM is more robust for r compared to Danino and vice versa. b) Heatmap for part a. thresholded at the value 0. Regions with negative values in a. have dark red color here, while regions with positive values in a. are colored blue. c) Heatmap for the effect sizes for individual points calculated using bootstrap estimates for the distribution for *r_Danino_* − *r_HOTFM_* Robustness Analysis for Rate of Synchronization (*r*). If *r_Danino_* − *r_HOTFM_* < 0, effect size is reported for greater robustness of HOTFM, while if *r_Danino_* − *r_HOTFM_* > 0, effect size (cohen’s *d*) is reported for greater robustness of Danino. The color scheme is as follows—Blue (negligible effect, *d* < 0.2), Cyan (small effect, 0.2 ≤ *d* < 0.5), Yellow (medium effect, 0.5 ≤ *d* < 0.8) and Dark Red (large effect, d ≥ 0.8). d) Heatmap for the presence or absence of 0 in the 95% bootstrapped confidence interval. Dark red means absence of 0 while blue represents presence.

**Fig 10:**
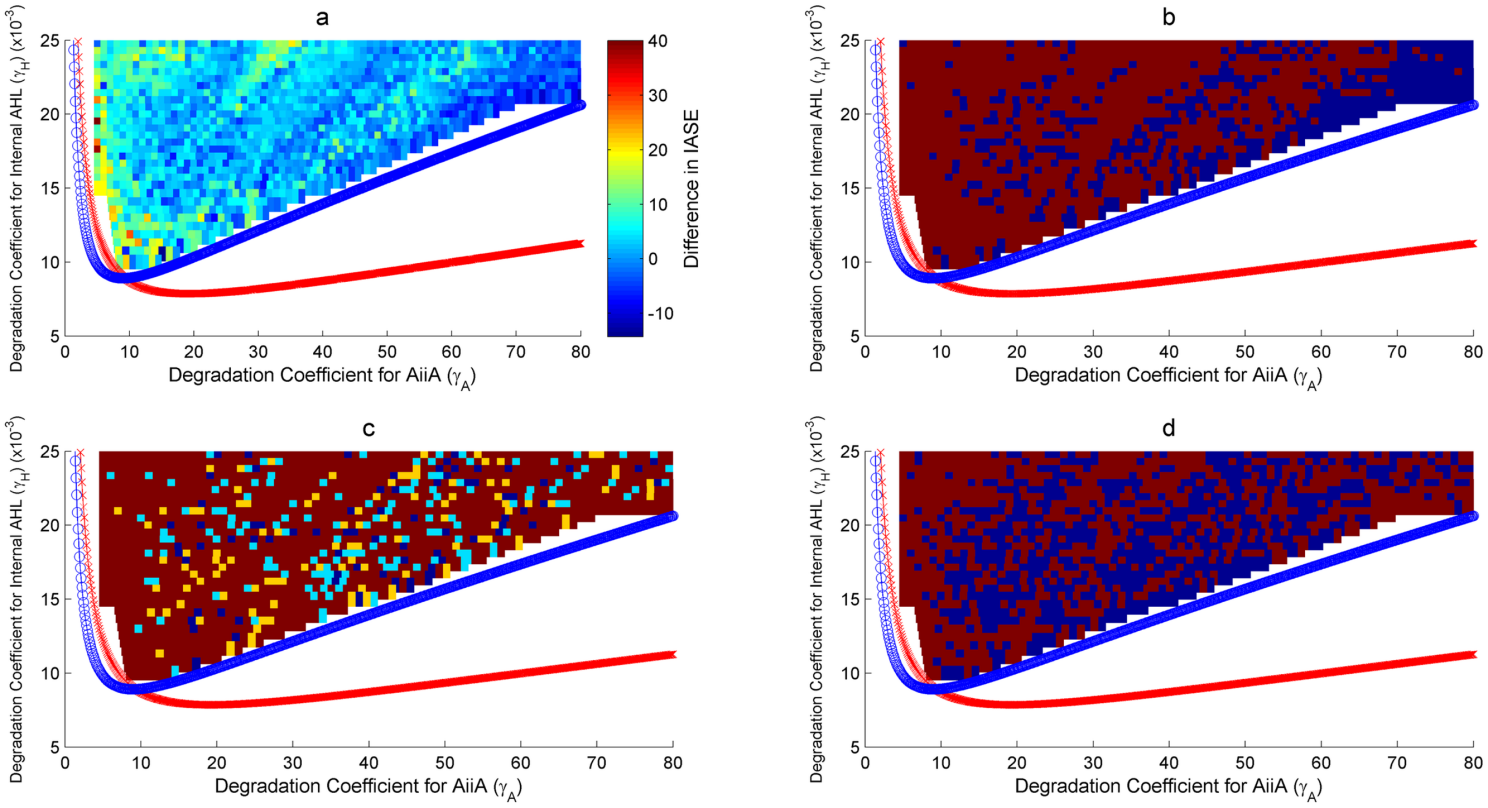
Robustness analysis for IASE in the *γ_H_*-*γ_A_* parameter space. Robustness analysis with respect to Integrated Absolute Synchronization Error (IASE). Two parameter bifurcation plots are presented for HOTFM and the Danino oscillators in the *γ_H_*-*γ_A_* parameter space for IASE. The blue (red) curve respresents the hopf branch for HOTFM (Danino). Region above the hopf branch corresponds to parameter values where oscillations occur, while for regions below the hopf branch oscillation dies and steady state solution arises. For a comparative analysis of robustness between HOTFM and Danino we consider 1510 points in the region of oscillations common to both HOTFM and Danino. At each point the system of equations (containing a linear array of 6 cells for both HOTFM and Danino) is numerically integrated for 500 minutes. From the obtained solutions IASE is calculated. For points which have *IASE_Danino_* − *IASE_HOTFM_* > 0, HOTFM is more robust compared to Danino, while for *IASE_Danino_* − *IASE_HOTFM_* < 0 Danino is more robust. a) Heatmap representing *IASE_Danino_* − *IASE_HOTFM_* at the 1510 points in the oscillatory region common between HOTFM and Danino. Positive values suggest that HOTFM is more robust for IASE compared to Danino and vice versa. b) Heatmap for part a. thresholded at the value 0. Regions with positive values in a. have dark red color here, while regions with negative values in a. are colored blue. c) Heatmap for the effect sizes for individual points calculated using bootstrap estimates for the distribution for *IASE_Danino_* − *IASE_HOTFM_* Robustness Analysis for IASE. If *IASE_Danino_* − *IASE_HOTFM_* > 0, effect size is reported for greater robustness of HOTFM, while if *IASE_Danino_* − *IASE_HOTFM_* < 0, effect size (cohen’s *d*) is reported for greater robustness of Danino. The color scheme is as follows—Blue (negligible effect, *d* < 0.2), Cyan (small effect, 0.2 ≤ *d* < 0.5), Yellow (medium effect, 0.5 ≤ *d* < 0.8) and Dark Red (large effect, d ≥ 0.8). d) Heatmap for the presence or absence of 0 in the 95% bootstrapped confidence interval. Dark red means absence of 0 while blue represents presence.

**Fig 11:**
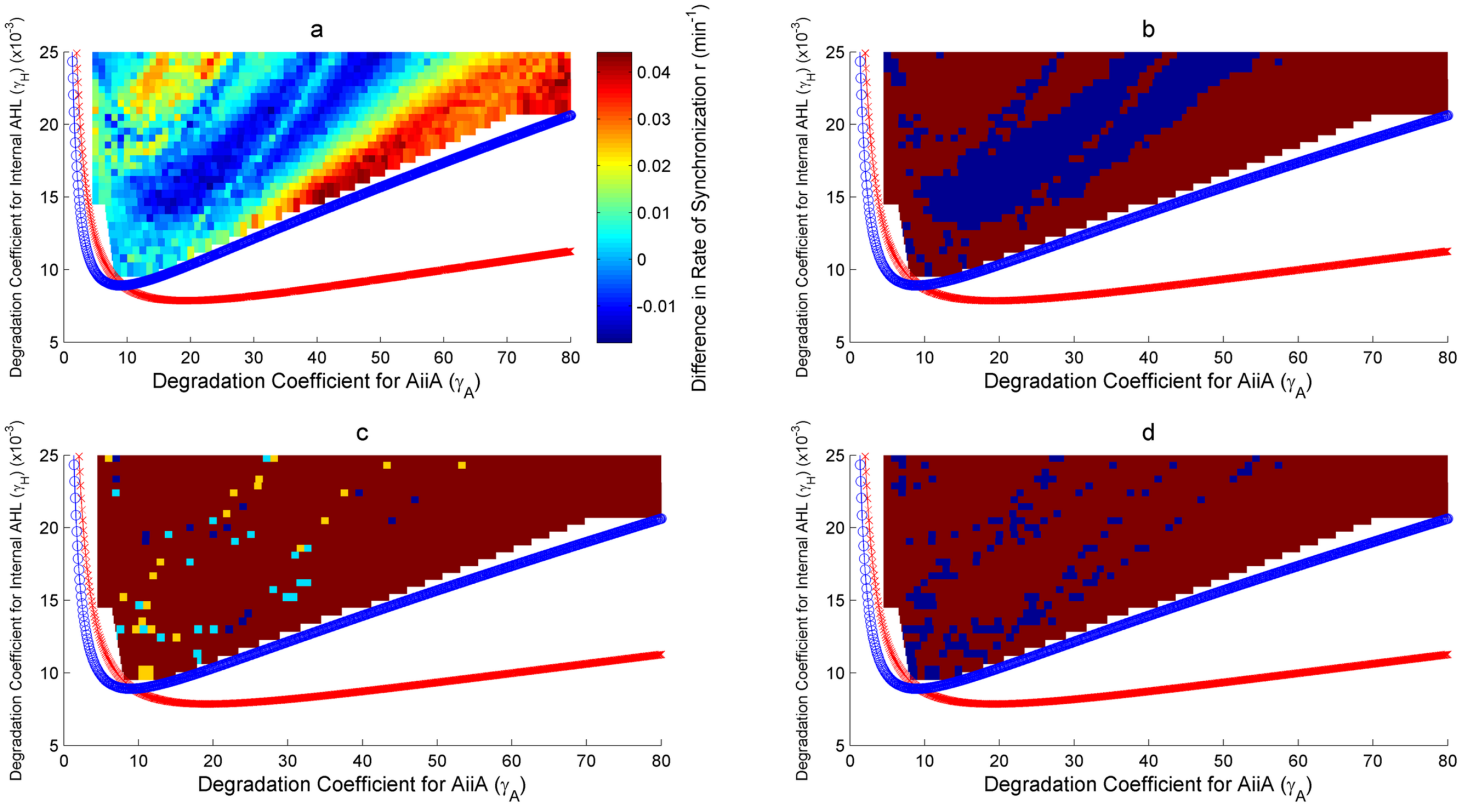
Robustness analysis for Rate of Synchronization (*r*) in the *γ_H_*-*γ_A_* parameter space. Robustness analysis with respect to Rate of Synchronization (*r*). Two parameter bifurcation plots are presented for HOTFM and the Danino oscillators in the *γ_H_*-*γ_A_* parameter space for *r*. The blue (red) curve respresents the hopf branch for HOTFM (Danino). Region above the hopf branch corresponds to parameter values where oscillations occur, while for regions below the hopf branch oscillation dies and steady state solution arises. For a comparative analysis of robustness between HOTFM and Danino we consider 1510 points in the region of oscillations common to both HOTFM and Danino. At each point the system of equations (containing a linear array of 6 cells for both HOTFM and Danino) is numerically integrated for 500 minutes. From the obtained solutions *r* is calculated. For points which have *r_Danino_* − *r_HOTFM_* < 0, HOTFM is more robust compared to Danino, while for *r_Danino_* − *r_HOTFM_* > 0 Danino is more robust. a) Heatmap representing *r_Danino_* − *r_HOTFM_* at the 1510 points in the oscillatory region common between HOTFM and Danino. Negative values suggest that HOTFM is more robust for r compared to Danino and vice versa. b) Heatmap for part a. thresholded at the value 0. Regions with negative values in a. have dark red color here, while regions with positive values in a. are colored blue. c) Heatmap for the effect sizes for individual points calculated using bootstrap estimates for the distribution for *r_Danino_* − *r_HOTFM_* Robustness Analysis for Rate of Synchronization (*r*). If *r_Danino_* − *r_HOTFM_* < 0, effect size is reported for greater robustness of HOTFM, while if *r_Danino_* − *r_HOTFM_* > 0, effect size (cohen’s *d*) is reported for greater robustness of Danino. The color scheme is as follows—Blue (negligible effect, *d* < 0.2), Cyan (small effect, 0.2 ≤ *d* < 0.5), Yellow (medium effect, 0.5 ≤ *d* < 0.8) and Dark Red (large effect, d ≥ 0.8). d) Heatmap for the presence or absence of 0 in the 95% bootstrapped confidence interval. Dark red means absence of 0 while blue represents presence.

As discussed in Assessment of the proposed Synthetic Circuits, two two-parameter bifurcations have been looked at for the robustness analysis. Figs 8, 9 and Figs 10, 11 show the bifurcation plots for HOTFM and the Danino oscillators in the *γ_I_*-*γ_A_* space and *γ_H_*-*γ_A_* spaces respectively. The regions above the hopf branches correspond to the parameter ranges for which oscillations are possible. For a comparative analysis, the oscillatory region common to both HOTFM and the Danino oscillators have been considered. Tables 1, 2 and Tables 3, 4 show the aggregate robustness metrics for the bifurcation analysis for HOTFM and the Danino oscillators in the *γ_I_*-*γ_A_* space and *γ_H_*-*γ_A_* spaces respectively.

**Table 1:** Aggregate robustness metrics for IASE in the *γ_I_*-*γ_A_* parameter space.

IASE		
**Aggregate Analysis**	**HOTFM**	**Danino**
Robustness	434/520 (0.8346)	86/520 (0.1653)
Confidence Interval (95%)	(0.8019, 0.8653)	(0.1346, 0.1981)
Effect Size	17.2518	17.2518
**Individual Point Analysis**	**HOTFM**	**Danino**
Points without 0 in CI (95%)	311/434 (0.7166)	41/86 (0.4767)
Effect Size (Cohen’s d)		
Negligible (d < 0.2)	10/434 (0.0230)	8/86 (0.0930)
Small (0.2 ≤ d < 0.5)	15/434 (0.0345)	14/86 (0.1628)
Medium (0.5 ≤ d < 0.8)	21/434 (0.0484)	12/86 (0.1395)
Large (d ≥ 0.8)	388/434 (0.8940)	52/86 (0.6047)

Aggregate metrics for robustness for IASE in the *γ_I_*-*γ_A_* parameter space for HOTFM and Danino. The upper part shows the overall robustness metrics over the entire range of 520 points, while the lower part gives the division of individual points into different effect size categories.

**Table 2:** Aggregate robustness metrics for Rate of Synchronization *r* in the *γ_I_*-*γ_A_* parameter space.

IASE		
**Aggregate Analysis**	**HOTFM**	**Danino**
Robustness	1070/1510 (0.7086)	440/1510 (0.2914)
Confidence Interval (95%)	(0.6854, 0.7311)	(0.2689, 0.3146)
Effect Size	16.9163	16.9163
**Individual Point Analysis**		
Points without 0 in CI (95%)	660/1070 (0.6168)	184/440 (0.4182)
Effect Size (Cohen’s d)		
Negligible (d < 0.2)	47/1070 (0.0439)	30/440 (0.0682)
Small (0.2 ≤ d < 0.5)	73/1070 (0.0682)	63/440 (0.1432)
Medium (0.5 ≤ d < 0.8)	61/1070 (0.0570)	49/440 (0.1114)
Large (d ≥ 0.8)	889/1070 (0.8308)	298/440 (0.6773)

Aggregate metrics for robustness for *r* in the *γ_I_*-*γ_A_* parameter space for HOTFM and Danino. The upper part shows the overall robustness metrics over the entire range of 520 points, while the lower part gives the division of individual points into different effect size categories.

**Table 3:** Aggregate robustness metrics for IASE in the *γ_H_*-*γ_A_* parameter space.

Rate of Synchronization (r)		
**Aggregate Analysis**	**HOTFM**	**Danino**
Robustness	367/520 (0.7058)	153/520 (0.2942)
Confidence Interval (95%)	(0.6654, 0.7442)	(0.2558, 0.3346)
Effect Size	9.7242	9.7242
**Individual Point Analysis**		
Points without 0 in CI (95%)	256/367 (0.6975)	81/153 (0.5294)
Effect Size (Cohen’s d)		
Negligible (d < 0.2)	10/367 (0.0272)	11/153 (0.0718)
Small (0.2 ≤ d < 0.5)	7/367 (0.0190)	8/153 (0.0523)
Medium (0.5 ≤ d < 0.8)	17/367 (0.0484)	15/153 (0.098)
Large (d ≥ 0.8)	333/367 (0.9073)	119/153 (0.7778)

Aggregate metrics for robustness for IASE in the *γ_H_*-*γ_A_* parameter space for HOTFM and Danino. The upper part shows the overall robustness metrics over the entire range of 1510 points, while the lower part gives the division of individual points into different effect size categories.

**Table 4:** Aggregate robustness metrics for Rate of Synchronization *r* in the *γ_I_*-*γ_A_* parameter space.

Rate of Synchronization (r)		
**Aggregate Analysis**	**HOTFM**	**Danino**
Robustness	1008/1510 (0.6675)	502/1510 (0.3324)
Confidence Interval (95%)	(0.6437, 0.6913)	(0.3086, 0.3563)
Effect Size	13.3888	13.3888
**Individual Point Analysis**		
Points without 0 in CI (95%)	930/1008 (0.9226)	432/502 (0.8606)
Effect Size (Cohen’s d)		
Negligible (d < 0.2)	11/1008 (0.0109)	3/502 (0.0059)
Small (0.2 ≤ d < 0.5)	10/1008 (0.0099)	10/502 (0.0199)
Medium (0.5 ≤ d < 0.8)	12/1008 (0.0119)	12/502 (0.0239)
Large (d ≥ 0.8)	975/1008 (0.9673)	477/502 (0.9502)

Aggregate metrics for robustness for *r* in the *γ_H_*-*γ_A_* parameter space for HOTFM and Danino. The upper part shows the overall robustness metrics over the entire range of 1510 points, while the lower part gives the division of individual points into different effect size categories.

#### Robustness analysis for IASE

**Fig 12:**
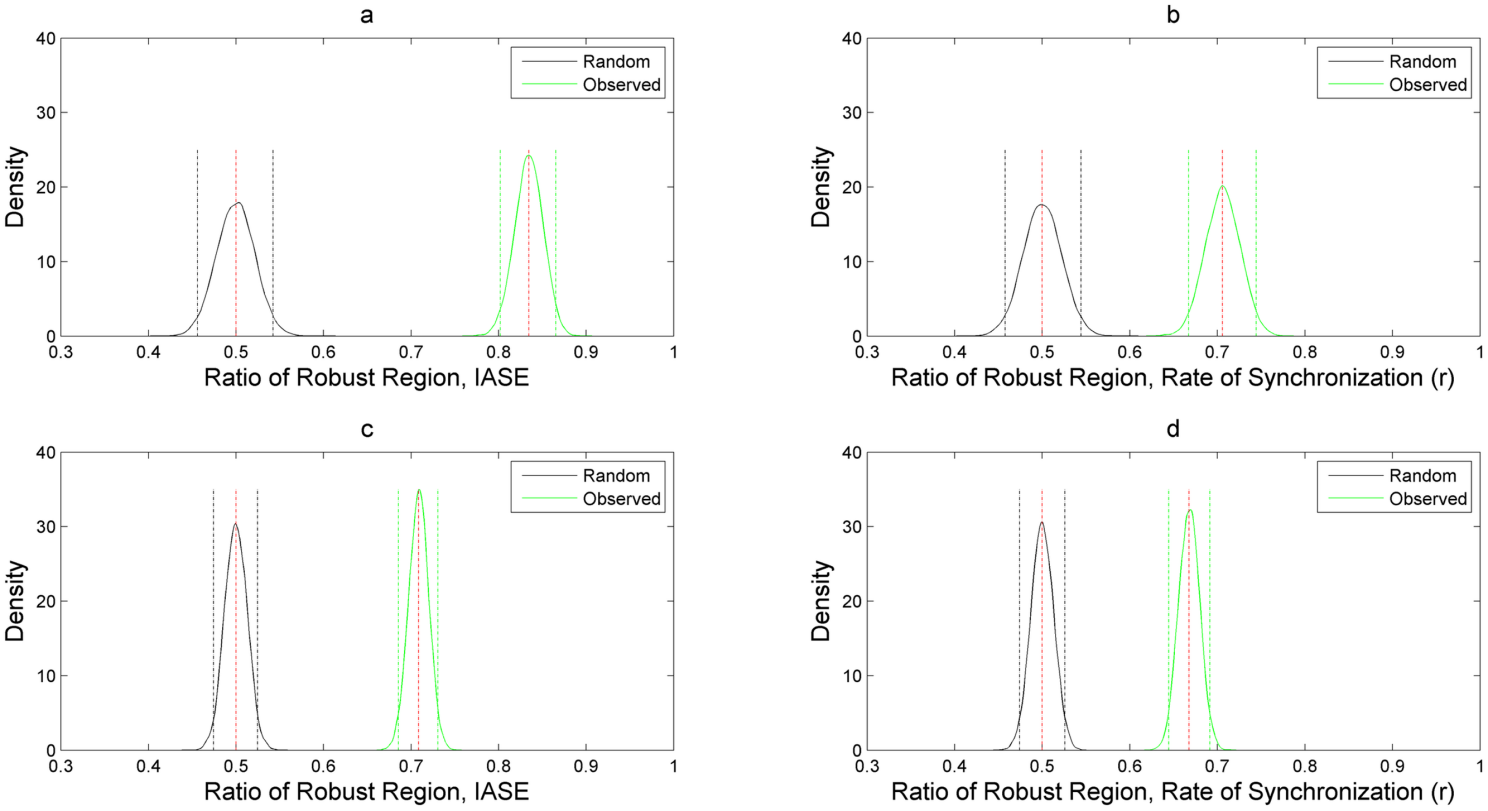
Distribution of ratio of robust region for IASE and *r* compared against random assignment of robustness. Probability density plots for comparing the ratio of number of points for which HOTFM is more robust compared to Danino for IASE and *r* against random assignment of robustness. The distribution for For the observed ratio of the number of points where HOTFM is more robust are obtained using boostrap sampling with replacement repeated 1000 times. For the density for the random case, bootstrap samples are drawn 1000 times assuming that both HOTFM and Danino are equally likely to be more robust than the other topology. a) Probability density plots for IASE for the observed and random case in the *γ_I_*-*γ_A_* parameter space. b) Probability density plots for *r* for the observed and random case in the *γ_I_*-*γ_A_* parameter space. c) Probability density plots for IASE for the observed and random case in the *γ_H_*-*γ_A_* parameter space. d) Probability density plots for *r* for the observed and random case in the *γ_H_*-*γ_A_* parameter space.

It is evident from Figs 8, 10 and Tables 1, 3 that on an aggregate basis HOTFM is more robust compared to the Danino oscillator. Among the 520 (1510) points explored in the *γ_I_*-*γ_A_* (*γ_H_*-*γ_A_*) parameter space, for 434 (1070) points (*IASE_Danino_ − IASE_HOTFM_*) > 0 and HOTFM is more robust, while for 86 (440) points (*IASE_Danino_ − IASE_HOTFM_*) < 0 and the Danino oscillator is more robust. To gauge the extent to which these estimates differ from randomly assigning robustness attribute (whether HOTFM is more robust or Danino) to the 520 (1510) points, boostrap resampling was conducted to generate the distributions as shown in Fig 12. The corresponding confidence intervals and effect sizes (cohen’s d) [52] are displayed in Tables 1 and 3. The 17.25 (16.91) value of cohen’s d suggests an extremely large deviation from the case of random assignment [52].

**Fig 13:**
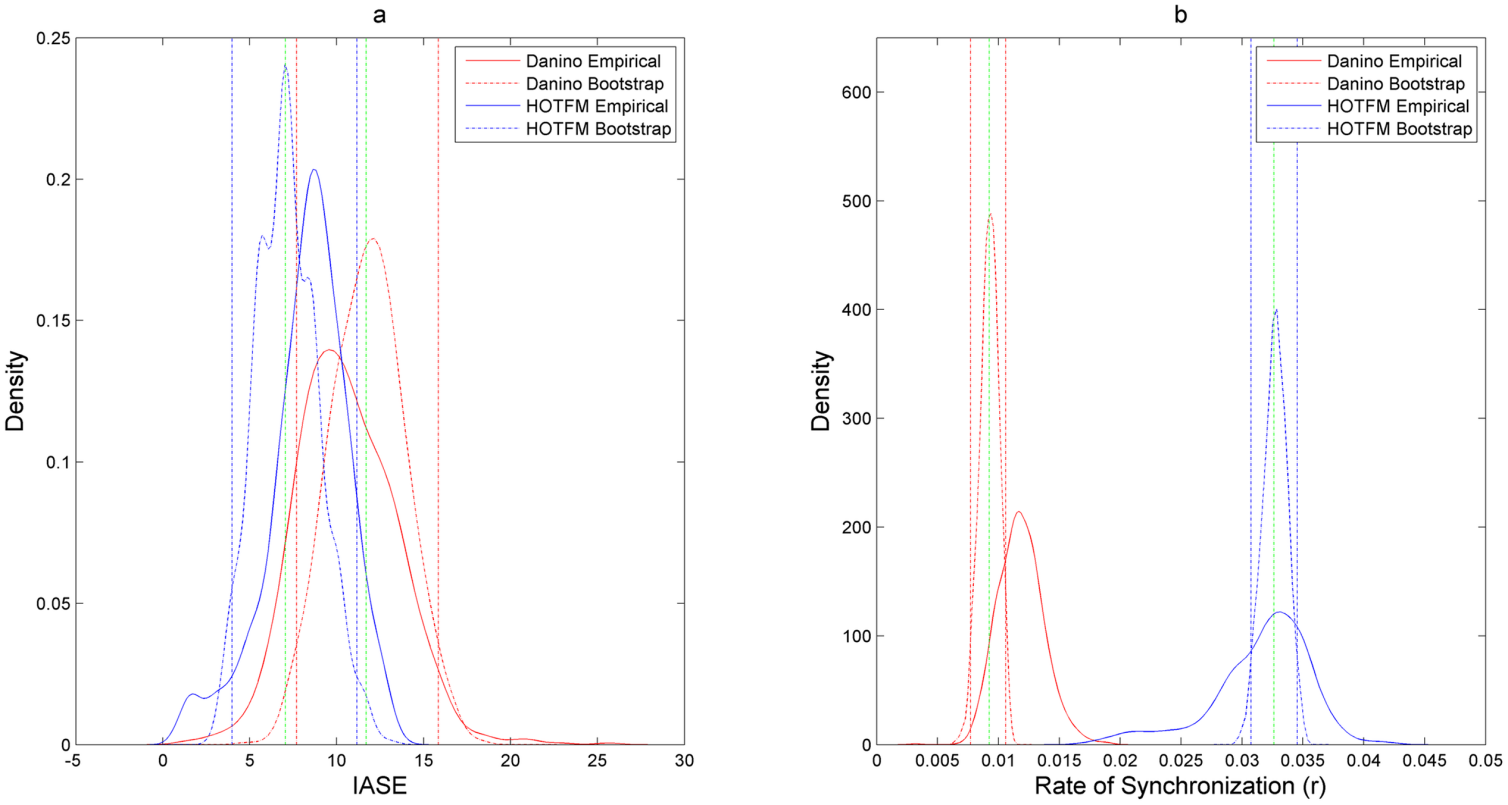
Bootstrap distributions (dotted curves) of IASE and *r* for indivudual points for Danino (red) and HOTFM (blue). **Actual distributions (solid curves) generated by the numerically integarting the system 1000 times are also shown for** Plots of bootstrap probability distributions for IASE and *r* for Danino (red) and HOTFM (blue). Green lines correspond to the observed values while the other dotted lines represent the 95% confidence intervals. a) Distributions for IASE. b) Distributions for (r).

Boostrap estimates can also be generated for individual points in the parameter space Fig 13; for a given point in the parameter space, IASE values for the cells in the colony are sampled with replacement a 1000 times and for each such sample, the new IASE values are aggregated, thus giving the bootstrap distribution. If {*IASE_i_* ∣ 1 ≤ *i* ≤ *n*} represents the IASE values for each cell with respect to its immediate neighbour on the right, for each bootstrap new IASE values are obtained after sampling with replacement from these observed IASE values for the cells. The boostrap distributions for HOTFM and the Danino oscillators can be compared for all the points in the parameter space, giving the effect sizes for individual points Fig 8. It is evident that majority of the points have large effect sizes for the difference in IASE (positive or negative) between HOTFM and the Danino oscillators.

#### Robustness analysis for Rate of Synchronization (*r*)

It is evident from Figs 9, 11 and Tables 2, 4 that on an aggregate basis HOTFM is more robust compared to the Danino oscillator for synchronization rate as well. Among the 520 (1510) points explored in the *γ_I_*-*γ_A_* (*γ_H_*-*γ_A_*) parameter space, for 367 (1008) points (*r_Danino_ − r_HOTFM_*) < 0 and HOTFM is more robust, while for 153 (502) points (*r_Danino_ − r_HOTFM_*) > 0 and the Danino oscillator is more robust. To gauge the extent to which these estimates differ from randomly assigning robustness attribute (whether HOTFM is more robust or Danino) to the 520 (1510) points, boostrap resampling was conducted to generate the distributions as shown in Fig 12. The corresponding confidence intervals and effect sizes (cohen’s d) [52] are displayed in Tables 2 and 4. The 9.72 (13.39) value of cohen’s d suggests an extremely large deviation from the case of random assignment [52].

Similar to IASE, boostrap estimates can again be generated for individual points in the parameter space Fig 13; for a given point in the parameter space, *r* values for the cells in the colony are sampled with replacement a 1000 times and for each such sample, the new *r* values are aggregated, thus giving the bootstrap distribution. If {*r_i_* ∣ 1 ≤ *i* ≤ *n*} represents the *r* values for each cell with respect to its immediate neighbour on the right, for each bootstrap new *r* values are obtained after sampling with replacement from these observed *r* values for the cells. The boostrap distributions for HOTFM and the Danino oscillators can be compared for all the points in the parameter space, giving the effects sizes for individual points Fig 8. It is evident that majority of the points have large effect sizes for the difference in IASE (positive or negative) between HOTFM and the Danino oscillators.

### HOTFM: Generation of beats

**Fig 14:**
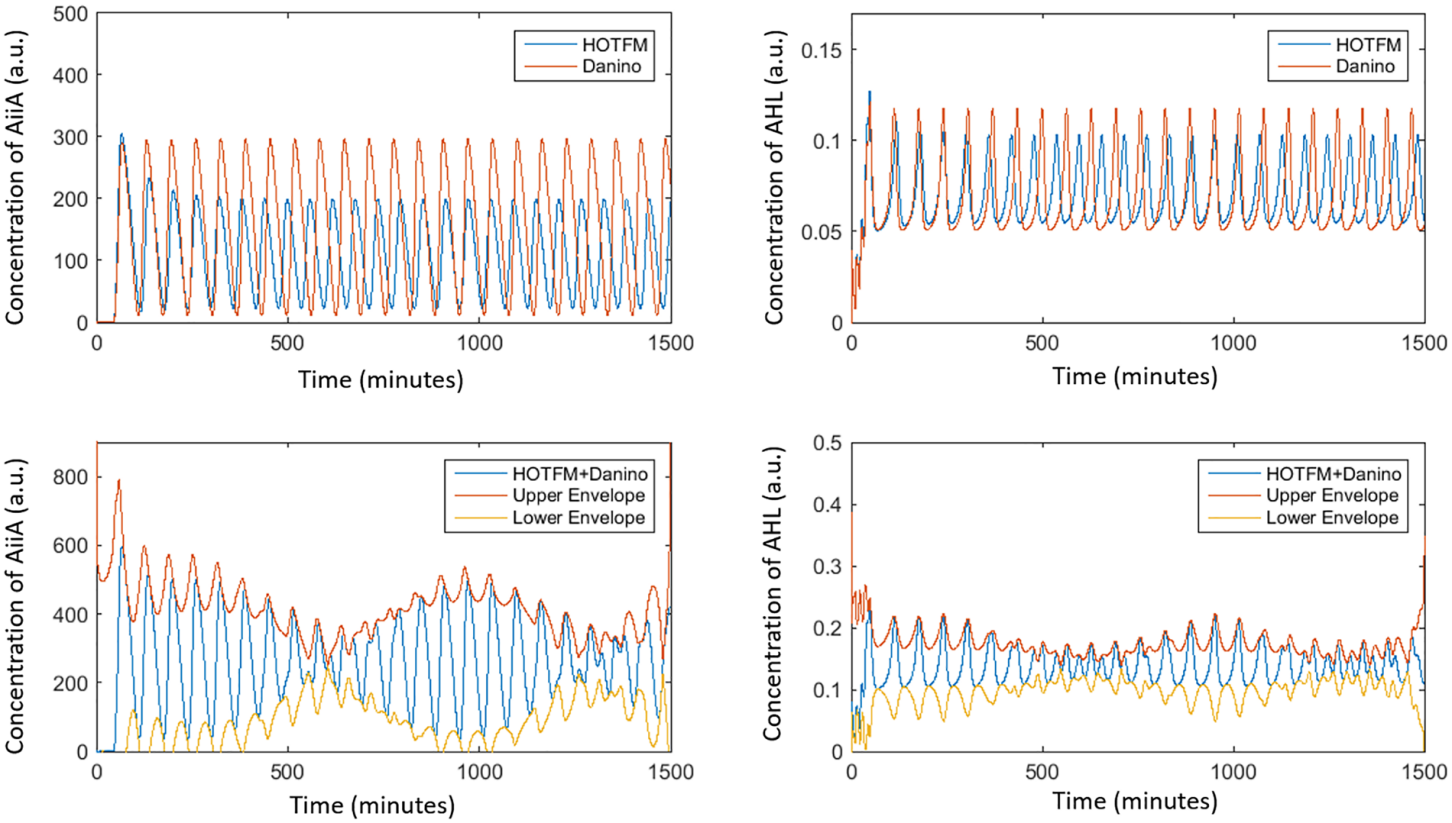
Generation of beats. When two colonies belonging to the Danino oscillator and oscillating HOTFM are mixed, we might get beats at the interface, if the HOTFM is in a state where it has slightly higher frequency of oscillation Here we see the aggregation of AiiA and external AHL signls from the two oscillators at their interface. The upper panels of the plot show the concentration of AiiA and AHL for cell numbers 100 and 101 (on either side of the interface) over time. The lower panels show the interference pattern caused due to the interference of these two waveforms at the interface (between cell nos. 100 and 101). Thus the lower left panel shows what the concentration profile would have been, had there been an AiiA molecule present at the interface of the two cells. The lower right panel simply shows the external AHL concentration. Both of these show beat like patterns over time.

Fig 14 shows beats being generated when two colonies of the Danino oscillator and the HOT-FM are mixed. Our simulations are run in a linear array of 200 E.coli cells, of which the first 100 cells have the Danino circuitry, while the other 100 have the HOTFM circuit. In this experiment, the values for the parameters are adapted from [23]; for the equations unique to HOTFM, Eqs 5 and 6, the hill function parameters for the two component system are adapted from [31] for CcaS-CcaR; TetR is taken as the repressor downstream of Ccas-CcaR and the degradation rate for TetR is assumed to be 1.5. Beats were generated when the concentration of the AHL at the interface of cell numbers 100 and 101 was plotted with time. Also, had there been an AiiA molecule at the interface, the time series for that has been plotted as well. This also shows beat like patterns. This could be achieved biologically by placing membranes between cells number 100 and 101, such that the cells would not mix, and therefore the 100 cells of Danino would not diffuse into the HOTFM area and vice versa, while the AHL molecules could diffuse through it. In this section bound by two membranes, we would observe the pattern of beats in the AiiA and AHL levels S5 Video.

## Conclusion

Here we have computationally demonstrated a framework for reconfigurable circuits, by providing the simulations for a system which can generate responses allowing us to switch between three qualitative behaviors. The first behavior is of the native Danino oscillator, while the second is of a frequency modulation of the oscillator (HOTFM), and the third is one that drives out the oscillations and forces the system to a steady state constant response.

We have chosen light directed self-repression due to the noninvasive, fast acting and orthogonality of optogenetic systems [34, 53, 54]. We have also shown that, by modulating the strength of the degradation tag used [50], we can transform the output response obtained from the system for the same repressor, which also makes sense intuitively, as increasing the degradation rate of the repressor would require a higher strength of repressor to achieve the response that would ideally be done by a weaker repressor. This work is novel, as frequency modulation being shown for the first time in a biological oscillator. Another quality is that the system has cell—cell communication and is therefore synchronised, which is a highly desirable quality when we begin to talk of real world applications of these circuits in fields such as that of time dependent targeted drug delivery, as we want to observe phenomenon at a bulk level. The requirement of synchronisation has seen recent developments in previous oscillators such as the repressilator achieving synchronisation as well [55]. Further, HOTFM exhibits more robust behavior in response to input perturbations with respect to synchronization in the transient phase.

Thus, here we have laid the framework for future work to be done in the field, which could have long range applications in the area of time dependent drug delivery, as the field advances and grows.

## Supporting information

S1 Video Average AiiA concentration without synchronization. The video shows the variation of AiiA for four cells over time, with the diffusion term set to zero (*D*1 = 0), implying no synchronization. As can be seen, the cells oscillate completely out of phase with each other, with the average oscillating with a very small amplitude. Simulations were run for a linear array of 200 Danino cells.
(MP4)

S2 Video AiiA concentration with synchronization. The synchronization process shown via generation of videos using MATLAB. Simulation video showing the variation of AiiA for four cells with synchronization (*D*1 = 100). It can be seen that with time, AiiA gets into synchrony, with the oscillations coming in phase, and the average also starts to oscillate in the same phase. Simulations were run for a linear array of 200 Danino cells.
(MP4)

S3 Video Frequency histogram for AiiA without synchronization. Frequency histogram for Danino cells without synchronization. At any time point, the number of cells at each concentration value of AiiA stays more or less uniform, indicating that even though oscillations are happening, the cells are not synchronized. Simulations were run for a linear array of 200 Danino cells.
(MP4)

S4 Video Frequency histogram for AiiA with synchronization. Frequency histogram for Danino cells with synchronization (*D*1 = 100). In this case, the cells start to synchronise, due to which we see a peak in the frequency distribution, which oscillates with time.
(MP4)

S5 Video Generation of beats. Representation of beats in time. The x-axis corresponds to the cell number and the color shows the concentration for AiiA. Blue shows low concentration and yellow high concentrations. The 100 cells on the left belong to HOTFM while the remaining 100 on the right to Danino. Initially, only middle cells for each colony have non-zero concentration and as time progresses, diffusion of AHL spreads the oscillation throughout the mix. We get beats at the interface of the two colonies.
(MP4)

S1 Fig Reconfigurability in a simple circuit. Simplification of (a) The two component Danino Oscillator in the native state (Repression by C not active) and (b) Re configuring of the oscillator into an oscillation kill switch, by repression of B by C.
(TIF)

S1 Equation Equation modifications for oscillation killing behavior. 1.1—Modified differential equation for aiiA, with a hill function based repression of aiiA; 1.2—Differential equation for the repressor (CI).
(TIF)

S2 Fig Simulation for the simple oscillation kill switch (LuxI). Concentration of AiiA as a function of time. The system is kept repression free for t < 200 min. At t = 200 mins, the repression is turned on, which quickly causes the oscillations to stop, and sends the AiiA levels in all of the cells to a near zero value. The thick yellow line depicts the mean concentration of aiiA across all the cells at any time.
(PNG)

S3 Fig Simulation for the simple oscillation kill switch (AiiA). Levels of Lux I. Again, the repression is turned on at t = 200 mins, and we see the oscillations ceasing and the levels of LuxI going up constantly, as the production rate now becomes higher than the rate of degradation. Ideally, due to toxicity, the LuxI levels would flatten out to a maximum at a certain point of time.
(PNG)

S4 Fig Proposed circuit for the temperature oscillation kill switch. Proposed circuit and mode of action for the reconfigurable oscillation kill switch—oscillator.
(PNG)

S5 Fig Logic circuit for the temperature dependent oscillation kill switch. Logic circuit for the proposed oscillation kill switch—oscillator (Created using logicly, https://logic.ly).
(PNG)

S6 Fig Heatmap for the concentration of AiiA for HOTFM and Danino oscillators. Heatmap for the concentration of AiiA as a function of time. The x-axis represents the cell number for which AiiA concentration is being measured, while the y-axis shows the time in minutes. The top panel shows the heatmap for the Danino while the lower for the HOTFM oscillators respectively. The scale varies from blue to yellow, thus blue corresponds to lower concentration while yellow to high concentrations. Here, we see that the yellow regions for Danino are brighter than for HOTFM. However, HOTFM shows a smaller spacing between two yellow bands (peaks) compared to Danino. There are 100 cells each for Danino and HOTFM oscillators.
(PNG)

S7 Fig Logic circuit for HOTFM. Logic circuit for the proposed HOTFM optogenetically tunable frequency modulating oscillator (Created using logicly, https://logic.ly).
(PNG)

S1 Code Matlab scripts. The Matlab scripts used for conducting the numerical bifurcation and time intergation analyses. A Readme file with instructions on using these scripts has been included as well.
(GZ) Click here for additional data file.

S1 File Supporting information details. This file contains further details for the various supporting information figures and videos. Specifically, details regarding the synchronized behavior of the Danino oscillator, S1–S4 Videos, have been given. Further, the oscillation kill switch depicted in S1 Fig has been explained in some detail.
(PDF)
