# Supplementary figures and images for: A novel optogenetically tunable frequency modulating oscillator
Source: PLoS One. 2018 Feb 1;13(2):e0183242. doi: 10.1371/journal.pone.0183242 (PMC5794059; doi:10.1371/journal.pone.0183242)

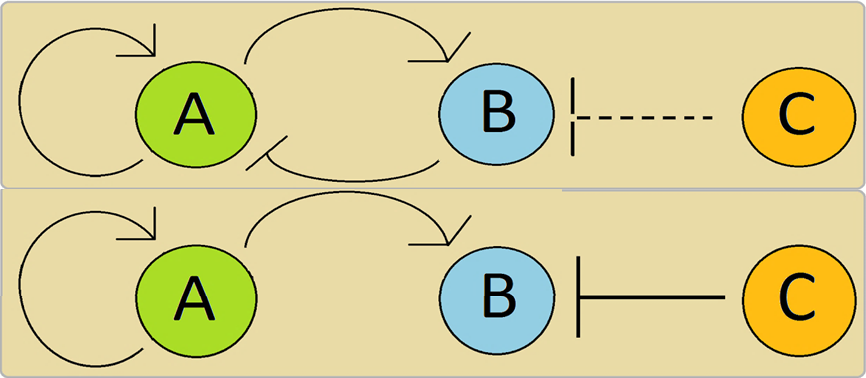

Supplement: S1 Fig — Simplification of (a) The two component Danino Oscillator in the native state (Repression by C not active) and (b) Re configuring of the oscillator into an oscillation kill switch, by repression of B by C. (TIF) [file pone.0183242.s006.tif]

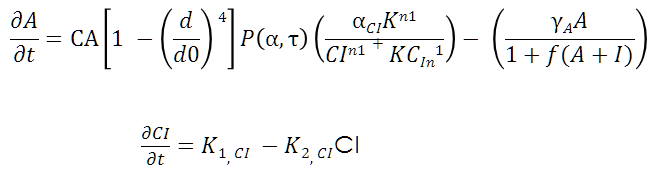

Supplement: S1 Equation — 1.1—Modified differential equation for aiiA, with a hill function based repression of aiiA; 1.2—Differential equation for the repressor (CI). (TIF) [file pone.0183242.s007.tif]

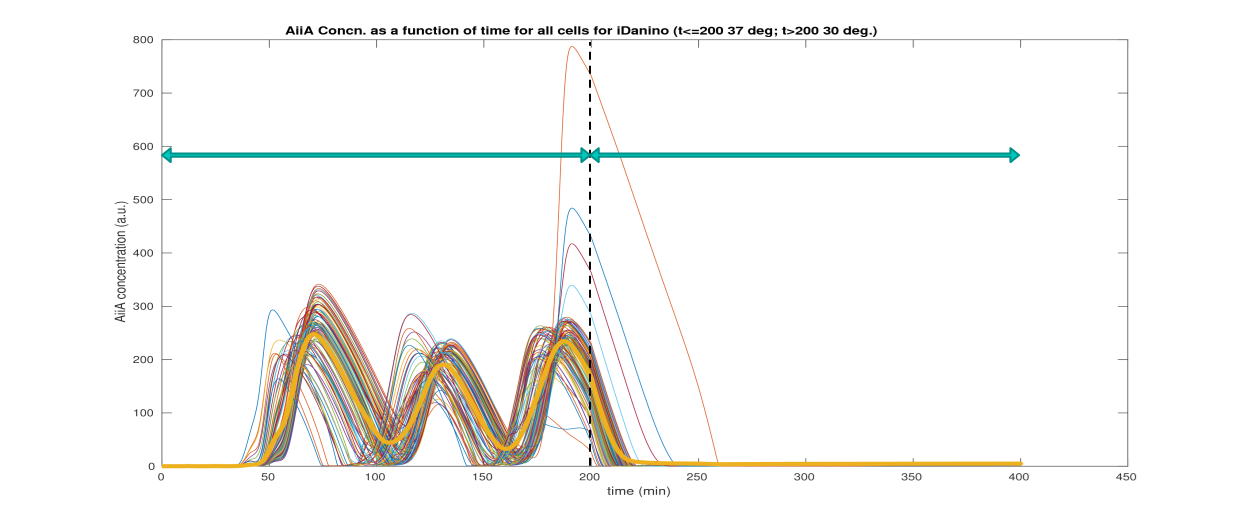

Supplement: S2 Fig — Concentration of AiiA as a function of time. The system is kept repression free for t < 200 min. At t = 200 mins, the repression is turned on, which quickly causes the oscillations to stop, and sends the AiiA levels in all of the cells to a near zero value. The thick yellow line depicts the mean concentration of aiiA across all the cells at any time. (PNG) [file pone.0183242.s008.png]

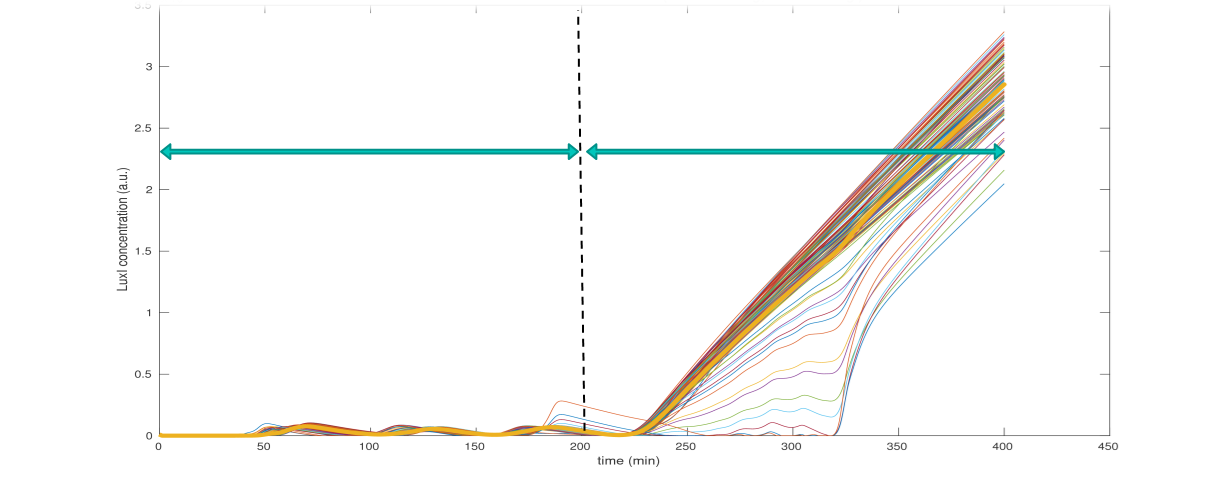

Supplement: S3 Fig — Levels of Lux I. Again, the repression is turned on at t = 200 mins, and we see the oscillations ceasing and the levels of LuxI going up constantly, as the production rate now becomes higher than the rate of degradation. Ideally, due to toxicity, the LuxI levels would flatten out to a maximum at a certain point of time. (PNG) [file pone.0183242.s009.png]

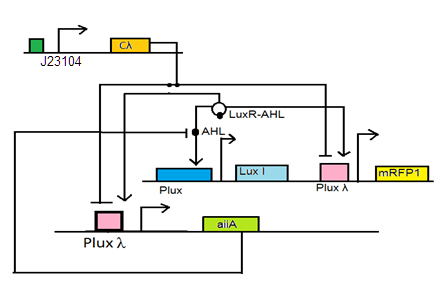

Supplement: S4 Fig — Proposed circuit and mode of action for the reconfigurable oscillation kill switch—oscillator. (PNG) [file pone.0183242.s010.png]

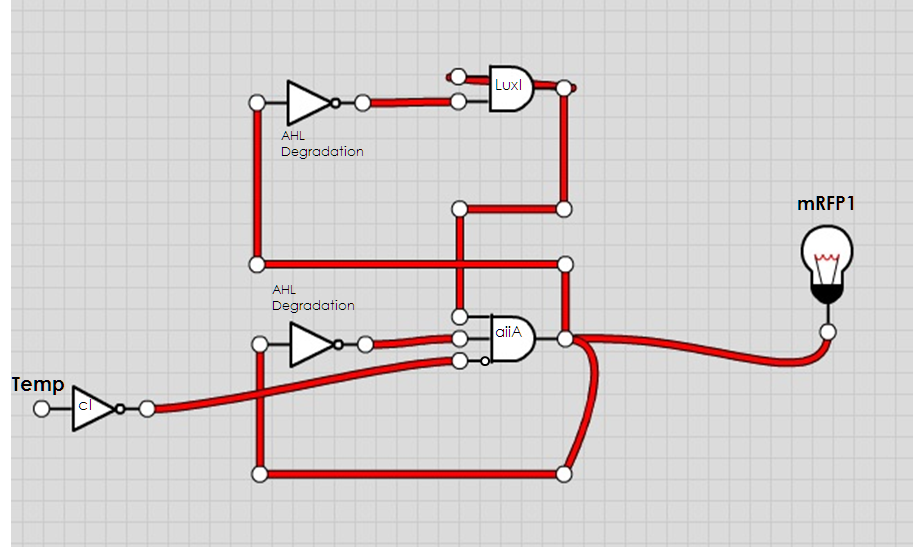

Supplement: S5 Fig — Logic circuit for the proposed oscillation kill switch—oscillator (Created using logicly, https://logic.ly). (PNG) [file pone.0183242.s011.png]

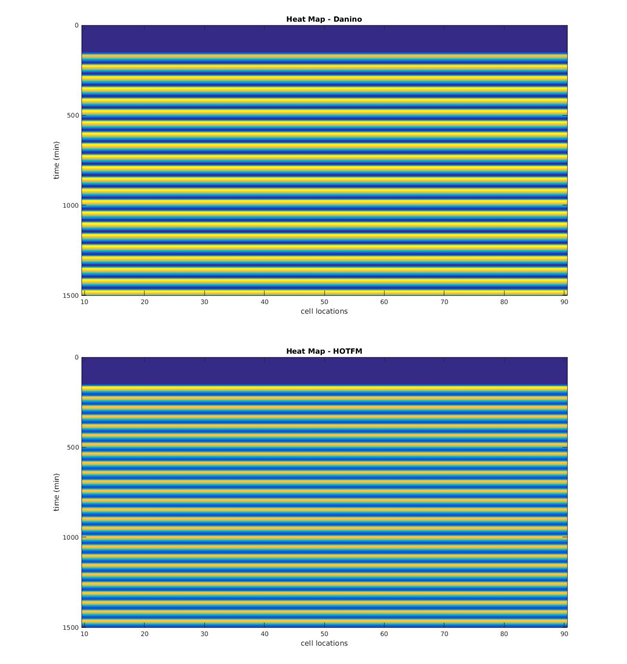

Supplement: S6 Fig — Heatmap for the concentration of AiiA as a function of time. The x-axis represents the cell number for which AiiA concentration is being measured, while the y-axis shows the time in minutes. The top panel shows the heatmap for the Danino while the lower for the HOTFM oscillators respectively. The scale varies from blue to yellow, thus blue corresponds to lower concentration while yellow to high concentrations. Here, we see that the yellow regions for Danino are brighter than for HOTFM. However, HOTFM shows a smaller spacing between two yellow bands (peaks) compared to Danino. There are 100 cells each for Danino and HOTFM oscillators. (PNG) [file pone.0183242.s012.png]

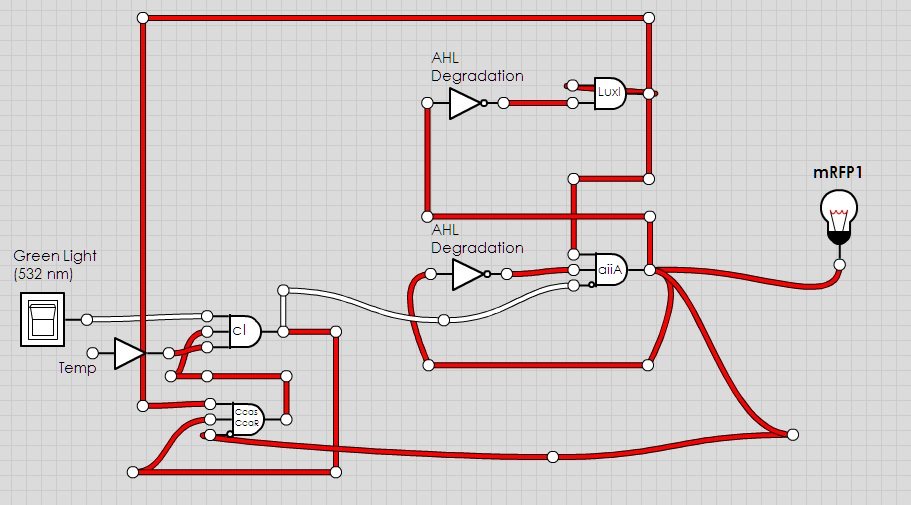

Supplement: S7 Fig — Logic circuit for the proposed HOTFM optogenetically tunable frequency modulating oscillator (Created using logicly, https://logic.ly). (PNG) [file pone.0183242.s013.png]
