# Supplementary material for: A novel optogenetically tunable frequency modulating oscillator
Source: PLoS One. 2018 Feb 1;13(2):e0183242. doi: 10.1371/journal.pone.0183242 (PMC5794059; doi:10.1371/journal.pone.0183242)
Supplement: S1 File — This file contains further details for the various supporting information figures and videos. Specifically, details regarding the synchronized behavior of the Danino oscillator, S1–S4 Videos, have been given. Further, the oscillation kill switch depicted in S1 Fig has been explained in some detail. (PDF) [file pone.0183242.s015.pdf]

In silico model for synchronization between cells in the Danino Oscillator

In order to show how the synchronized oscillator was different from a regular unsynchronized oscillator, we ran two sets of simulations for a linear array of 200 cells in MATLAB, one with the D1 value set equal to 0 (implying no synchronization between cells), and one with a numerical approximation for the second order partial differential term appearing in the equation for external AHL in the Danino oscillator equations with D1 set to a value of 100. Following this, we generated videos using a MATLAB video generator package. Videos S1 Video and S2 Video show the variation of AiiA levels for four different cells over time, and also the average of all 200 cells. We see that when there is no diffusion of AHL (D1 = 0), the four cells are oscillating out of phase with each other, and the average oscillation amplitude remains more or less constant throughout, whereas when we add the diffusion term, there is a synchronization of the phases of the cells, and the average also starts to oscillate in phase, indicating that there is an AHL induced cell-cell communication based synchronization between cells. Videos S3 Video and S4 Video show the same trend, and are frequency histograms of the simulations.

S1 Video-S4 Video – Synchronisation Videos. The synchronization process shown via generation of videos using the MATLAB video generator package. Simulations were run for an array of 200 cells. S1 Video – Simulation video showing the variation of AiiA for four cells over time, with the diffusion term set to zero (D1 = 0), implying no synchronization. As can be seen, the cells oscillate completely out of phase with each other, with the average oscillating with a very small amplitude. S2 Video – Simulation video showing the variation of AiiA for four cells with synchronization (D1 non zero). It can be seen that with time, the AiiA gets into synchrony, with the oscillations coming in phase, and the average also starts to oscillate in the same phase. S3 Video – Frequency histogram for cells without synchronization. At any time point, the number of cells at each concentration value of AiiA stays more or less uniform, indicating that even though oscillations are happening, the cells are not synchronized. S4 Video – Frequency histogram for cells with synchronization. In this case, the cells start to synchronize, due to which we see a peak in the frequency distribution, which oscillates with time.

Modifying the Danino oscillator makes it a reconfigurable oscillator with an oscillation kill switch.

We initially introduced reconfigurability into the Danino oscillator by trying to engineer it to exhibit oscillation kill behavior. For this, we decided to cut off the pathway that represses the LuxI part. Figure S1 shows a simplification of the native Danino oscillator, and the modification we made to it.

S1 Fig – Reconfigurability in a simple circuit Simplification of (a) The two component Danino Oscillator in the native state (Repression by C not active) and (b) Re configuring of the oscillator into an oscillation kill switch, by repression of B by C.

In the native state, the system works as the Danino oscillator, with A activating itself and B, while B represses A, leading to oscillations. In the second configuration, repression by part C becomes active, stopping the production of B, and thereby the repression of A due to it, leading to A going to a constant ON state, with levels of B going down to near zero values, corresponding to the OFF state, thereby making it an oscillation kill switch.

We modeled the system out, with the modified equation for the production of B (AiiA), and an additional equation for the repressor C, shown in S1 Equation.

S1 Equation – Equation modifications for oscillation killing behavior. 1.1 - Modified differential equation for AiiA, with a hill function based repression of AiiA; 1.2 - Differential equation for the repressor (CI)

Upon parameter tuning and figuring out optimal values of cooperativity and hill function constants for the repressor, it was found to closely resemble those of the lambda repressor. The modelling of the system for a linear array of 200 cells is shown in figures S2 and S3. The system was kept repression free for t < 200 min. At t = 200 mins, the repression was turned on, which quickly caused the oscillations to stop, and sends the AiiA levels in all of the cells to a near zero value, and the levels of LuxI going up constantly, as the production rate now becomes higher than the rate of degradation. Ideally, due to toxicity, the LuxI levels would flatten out to a maximum at a certain point of time.

S2 Fig.– Simulation for the simple oscillation kill switch (LuxI). Concentration of AiiA as a function of time. The system is kept repression free for t < 200 min. At t = 200 mins, the repression is turned on, which quickly causes the oscillations to stop, and sends the AiiA levels in all of the cells to a near zero value. The thick yellow line depicts the mean concentration of AiiA across all the cells at any time

S3 Fig. – Simulation for the simple oscillation switch (AiiA). Levels of Lux I. Again, the repression is turned on at t = 200 mins, and we see the oscillations ceasing and the levels of LuxI going up constantly, as the production rate now becomes higher than the rate of degradation. Ideally, due to toxicity, the LuxI levels would flatten out to a maximum at a certain point of time

Proposed Circuit for the reconfigurable oscillation killing behavior

After running the simulations and tuning the parameters, it was observed that the lambda cI repressor would be idea to be used as the part "C" in the circuit. Therefore, we propose a circuit here as shown in figure S4. Further, we use the heat sensitive mutant of the lambda repressor, which remains completely thermally denatured at 37°C, not interfering in the circuit. However, once we switch the temperature to 30°C, the cI repressor becomes active, and starts to repress AiiA production, which is under a hybrid promoter (lux inducible, lambda repressible), in order to create the oscillation kill switch. Thus, reconfigurability is engineered into the circuit, with temperature being the mode of control for the system's reconfiguration. Figure S5 shows the logic implementation of the circuit.

S4 Fig. – Proposed circuit for the temperature oscillation kill switch. Proposed circuit and mode of action for the reconfigurable oscillation kill switch - oscillator

S5 Fig. – Logic circuit for the temperature dependent oscillation kill switch. Logic circuit for the proposed oscillation kill switch - oscillator (Created using logicly, https://logic.ly)

HOTFM Implementation and Change in Frequency Compared to the Danino Oscillator

If we had hypothetical oscillators with identical parameters, then, in principle, starting with identical initial conditions would make synchronization redundant. However, for real oscillators there is variability in parameters across cells and this necessitates synchronization even with

identical initial conditions. Figure S6 shows the spatiotemporal heatmaps for the concentration of AiiA for Danino and HOTFM.

S6 Fig. – Heat Maps for Danino oscillator and HOTFM. Heat maps for (a) the Danino oscillator and (b) the HOT-FM oscillator respectively. The frequency is visibly more for the HOT FM as compared to the Danino oscillator.

Interference between the two waveforms: The Danino collides with the HOT-FM to create beats

S5 Video shows the phenomenon of beats, when the two waves collide with destructive interference, caused due to a difference in frequency

S5 Video – HOTFM plus Danino causes beats

Logic Circuit for Implementation of the HOTFM

S7 Fig. - Digital logic circuit for HOT FM (designed using logicly, https://logic.ly)
